# Tuning Hydrophilicity of Aluminum MOFs by a Mixed‐Linker Strategy for Enhanced Performance in Water Adsorption‐Driven Heat Allocation Application

**DOI:** 10.1002/advs.202301311

**Published:** 2023-05-13

**Authors:** Bao N. Truong, Daiane D. Borges, Jaedeuk Park, Ji Sun Lee, Donghui Jo, Jong‐San Chang, Sung June Cho, Guillaume Maurin, Kyung Ho Cho, U‐Hwang Lee

**Affiliations:** ^1^ Research Group for Nanocatalyst (RGN) and Chemical & Process Technology Division Korea Research Institute of Chemical Technology (KRICT) Gajeong‐Ro 141 Yuseong‐gu Daejeon 34114 Republic of Korea; ^2^ Department of Advanced Materials and Chemical Engineering University of Science and Technology (UST) Gajeong‐Ro 217 Yuseong‐gu Daejeon 34113 Republic of Korea; ^3^ Department of Chemistry Sungkyunkwan University Seobu‐Ro 2066, Jangan‐gu Suwon 16419 Republic of Korea; ^4^ ICGM, Univ. Montpellier, CNRS, ENSCM Montpellier 34095 France; ^5^ Institute of Physics Federal University of Uberlândia Uberlândia MG 38408‐100 Brazil; ^6^ Department of Chemical Engineering Chonnam National University Yongbong‐Ro 77, Buk‐gu Gwangju 61186 Republic of Korea

**Keywords:** low costs, metal–organic frameworks, mixed‐linkers, tuning hydrophilicity, water adsorption‐driven heat allocations

## Abstract

Water adsorption‐driven heat transfer (AHT) technology has emerged as a promising solution to address crisis of the global energy consumption and environmental pollution of current heating and cooling processes. Hydrophilicity of water adsorbents plays a decisive role in these applications. This work reports an easy, green, and inexpensive approach to tuning the hydrophilicity of metal–organic frameworks (MOFs) by incorporating mixed linkers, isophthalic acid (IPA), and 3,5‐pyridinedicarboxylic acid (PYDC), with various ratios in a series of Al−*x*IPA‐(100−*x*)PYDC (x: feeding ratio of IPA) MOFs. The designed mixed‐linkers MOFs show a variation of hydrophilicity along the fraction of the linkers. Representative compounds with a proportional mixed linker ratio denoted as KMF‐2, exhibit an S‐shaped isotherm, an excellent coefficient of performance of 0.75 (cooling) and 1.66 (heating) achieved with low driving temperature below 70 °C which offers capability to employ solar or industrial waste heat, remarkable volumetric specific energy capacity (235 kWh m^−3^) and heat‐storage capacity (330 kWh m^−3^). The superiority of KMF‐2 to IPA or PYDC‐containing single‐linker MOFs (CAU‐10‐H and CAU‐10pydc, respectively) and most of benchmark adsorbents illustrate the effectiveness of the mixed‐linker strategy to design AHT adsorbents with promising performance.

## Graphical abstract

A new series of metal–organic frameworks are synthesized by incorporating mixed isophthalic acid and 3,5‐pyridinedicarboxylic acid. Hydrophilicity of the materials is easily tuned by varying ratios of the two linkers. The representative compounds with proportional mixed linker ratio, denoted as KMF‐2, with enhanced performance, very low cost, and green synthesis are among the best candidates for water adsorption‐driven heat transfer application.

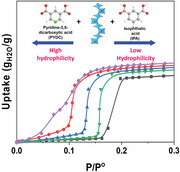

## 1 Introduction

Residential heating and cooling devices have accounted for a high share of global energy consumption.^[^ ^1^ ^]^ To achieve both minimizations of energy consumption and sustainability in line with a lowering of the dependency on fossil energy resources, the adsorption‐based heat transfer (AHT) systems consisting of adsorbent‐water working pair emerged as a promising alternative for cooling and heating purposes.^[^ ^2^, ^3^, ^4^, ^5^ ^]^ This system provides many advantages since a typical water working fluid has various decisive merits such as abundance, low cost, and environmental friendliness combined with high heat of evaporation (40.7 kJ mol^−1^). Further, these AHT systems can be operated at low temperatures (<80 °C) utilizing abundant, low‐temperature heat sources such as industrial waste or solar heat.^[^ ^6^ ^]^ Therefore, the development of effective water adsorbents for AHT systems have attracted enormous interest in past decades.^[^ ^7^, ^8^, ^9^, ^10^, ^11^, ^12^ ^]^

The traditional water adsorbents exhibit either very low hydrophilicity (e.g., silica gel) or high hydrophilicity (zeolites).^[^ ^13^, ^14^ ^]^ These features result in low‐temperature lift or high‐temperature sources needed to regenerate the working fluid. Another commercially available water adsorbent material, namely SAPO‐34, shows high performance in AHT application due to its suitable water adsorption characteristic,^[^ ^15^ ^]^ however, high desorption temperature is required (≈90 °C), making it inefficient to utilize low‐temperature and renewable heat sources. To overcome these limitations, metal–organic frameworks (MOFs) have been envisaged as a new class of water adsorbent owing to their high porosity, diversity in structure, and surface properties together with unprecedented chemical tunability.^[^ ^16^, ^17^, ^18^, ^19^ ^]^ Several MOFs such as Co‐CUK‐1, MIP‐200, CAU‐10, Aluminum Fumarate (MIL‐53‐FA), MIL‐160, CAU‐23, MOF‐303, CAU‐10pydc and KMF‐1^[^ ^6^, ^20^, ^21^, ^22^, ^23^, ^24^, ^25^ ^]^ have shown remarkable AHT performance with appropriate water adsorption features^[^ ^26^ ^]^ and regeneration at low temperature (< 80 °C). Among them, KMF‐1 and Co‐CUK‐1 have been demonstrated as record‐breaking water adsorbents in multi‐purpose applications for both adsorption‐driven cooling/chiller (AC) and adsorption‐driven heat pump (AHP), however, their synthesis costs are still too high for further promotion at the market. A water adsorbent for commercial use should satisfy all the following requirements: i) high working capacity (Δ*W*), ii) high coefficient of performance (COP) indicative of thermal efficiency, iii) fast kinetics of water adsorption and desorption, iv) regeneration with the use of renewable and natural heat sources, v) long‐term durability, specifically in hydrothermal conditions, i.e., steam condition, vi) scalable production under mild and green conditions along with high reaction yield, and vii) a cheap production cost.^[^ ^27^ ^]^

Even though CAU‐10 meets the majority of these criteria, its implementation in AHP system is hampered by its insufficient hydrophilicity. Systematic control of the MOF hydrophilicity without prominent reduction of its porosity is required to customize optimum material for a given water‐adsorption‐based application. A pioneering study very recently reported the preparation of a tailored series of Al‐based MOF water adsorbents for AHT application, named CAU‐10‐H‐IF via a mixed linker synthesis strategy using isophthalate (IPA) and 2,5‐furandicarboxylate (2,5‐FDC).^[^ ^28^ ^]^ These adsorbents were shown to exhibit distinct water adsorption features according to the mixed linker ratio, leading to an enhancement of the COP for cooling (COP_c_) compared to their respective bare adsorbents IPA‐based CAU‐10‐H and 2,5‐FDC‐based MIL‐160. However, the use of a non‐ecofriendly toxic solvent, *N,N*‐dimethylformamide (DMF) and a highly expensive linker, 2,5‐FDC, is critically disadvantageous for commercial use of these water sorbents in AHT system. Similarly, the mixed‐linker approach was successfully accomplished over the MOF‐303 structure enabling fine‐tuning of the surface hydrophilicity.^[^ ^29^, ^30^ ^]^ In another example, the attempt to prepare the mixed‐linker structure of CAU‐23 or MIL‐160 only resulted in the physically mixed phase form because of the mismatch of the linkers in a certain structure.^[^ ^31^ ^]^ Nevertheless, the linkers, such as 1H‐pyrazole‐3,5‐dicarboxylate (PYZDC), 2,4‐furandicarboxylate (2,4‐FDC), and 2,5‐thiophenedicarboxylate (2,5‐TDC) are still costly making the corresponding MOFs hardly transferable at the market.

Recently, CAU‐10H built by isophthalic acid (IPA) and CAU‐10pydc constructed by 3,5‐pyridinedicarboxylic acid (PYDC) were reported as practically viable water adsorbents due to their extremely low linker prices.^[^ ^32^ ^]^ Both MOFs also possess isostructure which favors the integration of the two linkers to a single phase of adsorbent. Furthermore, although CAU‐10H displays good performance in AC applications, this adsorbent is not applicable in AHP systems due to its poor performance in the working condition of these devices. Similarly, high hydrophilicity of CAU‐10pydc makes it a good candidate for AHP application but not suitable for AC use. Thus, the integration of the two linkers into one single structure can tune the hydrophilicity of the adsorbent for multi‐purpose AHT applications. For this purpose, herein, we devised an eco‐friendly green synthesis strategy of a new series of mixed‐linker Al‐MOFs incorporating cheap and commercially available organic linkers, IPA and PYDC. Basic properties of all obtained samples were characterized. The performance for AHT application of a new Al‐MOF constructed with a 50:50 mixed linker ratio denoted as KMF‐2 was evaluated and compared with the two neat MOFs and other benchmark MOFs as well. Deep understanding of the performance of KMF‐2 was also revealed using Grand‐Canonical Monte Carlo (GCMC) simulations.

## 2 Results and Discussion

### 2.1 Synthesis and Characterization

**Figure 1:**
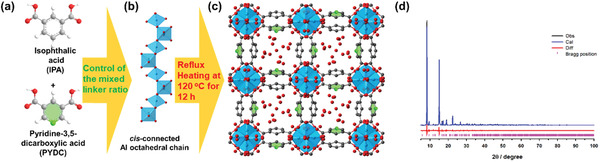
Schematic description for the synthesis of the mixed‐linker Al MOF KMF‐2 and its crystal structure determination. a) Organic linkers used for the mixed‐linker synthesis of KMF‐2 (equimolar ratio between isophthalate and 3,5‐pyridinedicarboxylate linkers). b) Helica chains cis‐connected Al octahedral by the µ‐OH and coordinated by four carboxylates from the linker along the *a*‐axis. c) Crystal structure of hydrated KMF‐2. d) Rietveld refinement of the KMF‐2 hydrated structure model. The experimental data are shown in black, simulation in blue, and difference in red. Allowed Bragg reflections are indicated as magenta ticks below. Color scheme: Al, blue; C, black; O, red; N, green (hydrogen atoms omitted for clarity).

Three different samples of Al−*x*IPA‐(100−*x*)PYDC [*x* = 25, 50, and 75, where x is the feeding ratio of IPA] were synthesized by varying the ratio of 3,5‐pyridinedicarboxylic acid (PYDC) and isophthalic acid (IPA), which are both cheap and commercially available linkers for the scalable production, under green synthesis and mild hydrothermal reflux condition at 120 °C (**Figure** **1**). Both CAU‐10pydc and CAU‐10‐H incorporating only PYDC and IPA linkers respectively were also prepared, as reference materials (see Experimental Section and Table S1 (Supporting Information) for further detail).^[^ ^20^ ^]^ The reaction yields and space‐time to yields for the three Al−*x*IPA‐(100−*x*)PYDC samples were achieved over 83% and 64.1 kg m^−3^ day^−1^, respectively. Powder X‐ray diffraction (PXRD) analysis was performed to evaluate the crystallinity of the three samples and determine their crystal structures. Their diffraction patterns showed highly intense Bragg peaks, being very similar to that of CAU‐10‐H and CAU‐10pydc (Figure S1, Supporting Information). The good crystallinity of these samples was further confirmed by scanning electron microscopy (SEM) images, presenting the microcrystals of the five Al−*x*IPA‐(100−*x*)PYDC samples with sizes under 8 µm (Figure S2, Supporting Information). To quantify the mixed linker ratio compared to those of feeding ratio, the three Al−*x*IPA‐(100−*x*)PYDC samples were examined by using ^1^H NMR spectroscopy after digestion by the solution of NaOD in D_2_O (Figure S3, Supporting Information). The linkers fractions of the synthesized samples were found to be very close to the feeding linkers ratios. This observation highlighted that the mixed‐linker approach allows the incorporation of two linkers into the isoreticular structure of CAU‐10‐H or CAU‐10pydc by controlling the linkers feeding ratio. The chemical formula of these compounds was further calculated using a combination of elemental analysis (EA) and inductively coupled plasma atomic emission spectroscopy (ICP‐AES) as illustrated in Table S2 (Supporting Information). The quantitative results of EA, ICP, and ^1^H NMR were all consistent, emphasizing a fine‐tuning of the mixed linker ratio in the prepared samples via our mixed‐linker synthesis approach.

To assess the porosity of all samples according to their mixed linker ratio, N_2_ and Ar adsorption isotherms were collected at 77 K and 87 K, respectively. Brunauer–Emmett–Teller surface area (*S*_BET_) determined from N_2_ adsorption isotherm was shown to increase by increasing the fraction of PYDC linker from 670 to 1030 m^2^ g^−1^ (Figure S4 and Table S3, Supporting Information). The pore size distributions (PSD) calculated by the Horvath–Kawazoe equation from the Ar adsorption isotherms (Figure S5a, Supporting Information), were found to be in the range of 0.55–0.6 nm (Figure S5b, Supporting Information).^[^ ^33^, ^34^ ^]^ Thermal stability of the samples was evaluated by both thermogravimetric analysis (TGA) and high‐temperature PXRD (HT‐PXRD) analysis (Figures S6 and S7, Supporting Information). All the samples displayed the distinct three weight losses in TGA curves with increasing temperature: weight loss of adsorbed water molecules (25−120 °C); decomposition of µ‐OH (380−440 °C) and decomposition of coordination bond of linkers (>450 °C).^[^ ^32^ ^]^ To crosscheck the thermal stability, one representative sample, denoted as KMF‐2, which was prepared with 50:50 PYDC/IPA ratio, was investigated by HT‐PXRD analysis up to 500 °C and was found to be thermally stable up to 350 °C. The hydrothermal and chemical stability were further tested over KMF‐2 under boiling water and a wide range of aqueous pH conditions (1 ≤ pH ≤ 12) for 24 h. The crystallinity and porosity of this sample after these treatments were maintained (Figure S8 and S9, and Table S3, Supporting Information) owing to its exceptional hydrothermal and chemical stability.

Structure analysis. To solve the structure of KMF‐2, Rietveld refinement was conducted using high‐resolution PXRD patterns for hydrated and dehydrated samples (**Figure** 1d, Figures S10 and S11, and Tables S4 and S5, Supporting Information). Four distinct structure models of KMF‐2 with 50:50 PYDC/IPA ratio corresponding to different possible distributions of the PYDC/IPA linker in the unit cell were first built in silico based on the dehydrated experimental structure and then, geometry optimized at the Density Functional Theory (DFT) level with the objective to identify if one configuration is more energetically stable compared to the others. The resulting DFT‐optimized structures and their corresponding total electronic energy, cell parameters, and textural properties are reported in Figure S16 and Table S6 (Supporting Information). Both the total electronic energy and the cell parameter of all these 4 structures are very similar with an energy difference of 7.8 kJ mol^−1^ from the less to the most stable structures. This observation clearly states that the position of PYDC does not impact the energetics of the MOF framework and therefore KMF‐2 is likely to be represented by any of these 4 structure models. We can also see that this linker distribution does not affect either the structural features or the textural properties of the resulting MOF (Table S6, Supporting Information).

**Figure 2:**
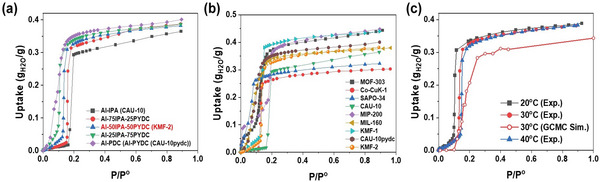
Water adsorption isotherms for mixed‐linker MOFs and benchmark adsorbents. a) Experimental water adsorption isotherms of the platform MOFs (CAU‐10 and CAU‐10pydc) and various mixed‐linker MOFs with different PYDC/IPA ratios at 30 °C (the GCMC simulated adsorption isotherm for KMF‐2 is also reported for comparison). b) Comparison of water adsorption isotherms of KMF‐2 and benchmark adsorbents at 30 °C. (c) Water adsorption isotherms of KMF‐2 at various temperatures (the GCMC simulated adsorption isotherm for KMF‐2 at 30 °C (red, open circle) is also presented for comparison).

Water Adsorption. The water adsorption isotherms of the three mixed linker samples as well as the reference CAU‐10‐H or CAU‐10pydc materials were measured at 30 °C. When increasing the PYDC /IPA linker ratio from 0 to 100%, the water sorption capacity was gradually increased from 0.29 to 0.35 g_H2O_ g_MOF_^−1^ at a relative pressure (*P*/*P*°) = 0.2, ascribed to the slightly different pore size of these samples (see PSD‐ Figure S5b, Supporting Information). Notably, their step‐like steep uptake of water was systematically shifted to lower *P*/*P* ^o^ by increasing the PYDC/IPA linker ratio (**Figure** **2**a). This observation highlights that the surface hydrophilicity of CAU‐10 structure can be finely controlled with simple reflux synthesis via the mixed‐linker synthesis approach. This is of interest since it is well documented that a fine‐tuning of surface hydrophilicity of water adsorbent plays a key role to achieve sustainability and high energy efficiency together with high performance in AHT devices.^[^ ^13^, ^14^, ^15^, ^28^ ^]^ Moreover, the water adsorption isotherms for all these samples only show one sharp step at a given *P*/*P*° supporting that the incorporation of the two linkers results in the formation of a single phase without the existence of physically mixed CAU‐10‐H and CAU‐10pydc phases in line with the PXRD structure analysis described above.^[^ ^28^ ^]^ Thus, the water adsorption performance of one representative sample (50:50 PYDC/IPA ratio), KMF‐2, was compared with other benchmarks reported as promising multi‐purpose water adsorbents in AHT application (**Figure** 2b). KMF‐2 shows intermediate water adsorption capacity compared with that of benchmarks in the *P*/*P*° ranges of 0.1–0.15. Importantly, its steep water uptake was observed in a very similar *P*/*P*° range to KMF‐1 and Co‐CUK‐1, which have been reported as exceptional multi‐purpose water adsorbents for AHT application, allowing utilization of low‐temperature heat sources below 70 °C.^[^ ^6^, ^25^ ^]^ The isosteric enthalpy of water adsorption (*Q*_st_) was derived by applying both Clausius–Clapeyron and Virial equations (Figure S12, Supporting Information)^[^ ^35^ ^]^ to the water isotherms collected at three different temperatures of 20–40 °C (**Figure** 2c). The *Q*_st_ values for KMF‐2 were found in the range of −52/−58 kJ mol^−1^, significantly higher than the heat of water evaporation (40.7 kJ mol^−1^).

### 2.2 Desorption Kinetic

Kinetic of water desorption of KMF‐2 and the two neat MOFs was evaluated by conducting TGA experiments of the saturated adsorbents under various heating rates (*β*).^[^ ^36^ ^]^ Derivative thermogravimetry (DTG) curves revealing the differentiation of percentage of weight loss per minute to temperature of the adsorbents are shown in Figure S13 (Supporting Information). Temperature at highest desorption rate under each heating rate was recorded as *T_d_* and used for Kissinger equation^[^ ^37^ ^]^ to assess the activation energy of desorption (*E*_d_). The data are included in Table S7 (Supporting Information). The *E*_d_ of the three adsorbents is in line with the trend of hydrophilicity of these materials. CAU‐10H has the lowest *E_d_* (83 kJ mol^−1^), following by KMF‐2 (*E_d_* = 88 kJ mol^−1^) and CAU‐10pydc (*E_d_* = 91 kJ mol^−1^). The results evidently support the success of mixed‐linker strategy in controlling the hydrophilicity of the adsorbent.

### 2.3 Evaluation of the Thermodynamic Performance in AHT Systems

**Figure 3:**
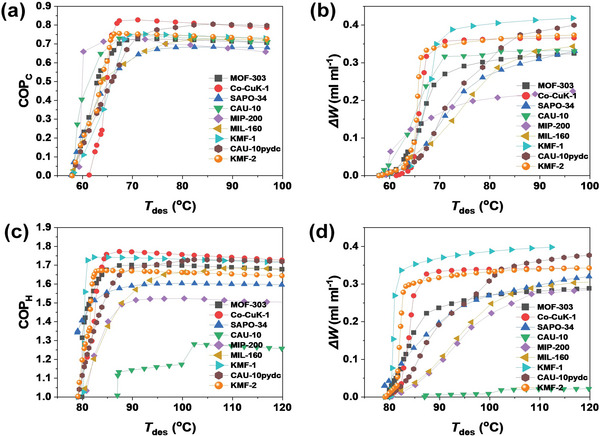
Thermodynamic evaluation of water adsorbents in AHT applications. a) Coefficient of performance in adsorptive cooling application (COP_C_) and b) volumetric working capacity of adsorptive cooling (AC) application at *T*_*ev*_ = 5 °C, *T*_*ads*_, and *T*_*con*_ = 30 °C according to *T*_*des*,_ and c) coefficient of performance in adsorption heat pump application (COP_H_) and d) volumetric working capacity of adsorptive heat pump (AHP) application at *T_ev_* = 15 °C*, T_con_* = 30 °C*, and T_ads_* = 45 °C according to *T*_*des*_.

The standard performance metrics for an adsorbent in a typical AHT application are its coefficient of performance for cooling (COP_C_) and heating (COP_H_), and its volumetric working capacity (*ΔW*) under various boundary conditions.^[^ ^38^ ^]^ One cycle of operation with a water/adsorbent working pair in AHT system consists of four thermodynamic equilibrium steps, controlled by the temperatures of evaporation (*T*_ev_), adsorption (*T*_ads_), condensation (*T*_con_), and desorption (*T*_des_) depending on cooling or heating purpose (Figure S14, Supporting Information).^[^ ^38^, ^39^ ^]^ Therefore, the performance evaluation of KMF‐2 was performed accordingly and the resulting values of COPs and Δ*W* were compared with that of the benchmark materials for both AC and AHP systems under various conditions (**Figure 3**; Figure S15, Supporting Information).^[^ ^25^ ^]^ The COP_C_ and Δ*W* in AC application were evaluated with the boundaries *T*_ev_ = 5 °C, *T*_ads_ = 30 °C and *T*_con_ = 30 °C as a function of *T*_des_ (**Figure** 3a,b). A COP_C_ of 0.75 was obtained for KMF‐2 with a low desorption temperature (*T*_des_<70 °C). This value is only slightly lower than that previously reported for Co‐CUK‐1 (0.83), and comparable to other benchmark MOFs (0.72–0.75). Besides, Δ*W* of KMF‐2 reaches 0.342 mL_H2O_ mL_MOF_^−1^ at *T*_des_ = 70 °C. This level of *ΔW* is comparable to that of the top‐ranked benchmark MOFs, KMF‐1 (0.358 mL_H2O_ mL_MOF_^−1^) and Co‐CUK‐1 (0.346 mL_H2O_ mL_MOF_^−1^), in AC application (**Figure** 3b). Regarding AHP application, the value of COP_H_ for KMF‐2 (1.66) is only slightly lower than Co‐CUK‐1 (1.76), KMF‐1 (1.74), and MOF‐303 (1.68), but its thermal efficiency is far above than that of SAPO‐34, MIL‐160, MIP‐200 and CAU‐10H at *T_ev_* = 15 °C, *T_con_* = 30 °C, *T_ads_* = 45 °C, and *T_des_* = 85 °C (**Figure** 3c). In these boundary conditions, *ΔW* of KMF‐2 (0.305 mL_H2O_ mL_MOF_^−1^) surpasses the values for all benchmark MOFs except KMF‐1 (0.352 mL_H2O_ mL_MOF_^−1^) (**Figure** 3d). Hence, KMF‐2 is highly expected to be a suitable water adsorbent for multi‐purpose use not only in AHT application but also in daily or seasonal heat storage and water harvesting in air owing to this exceptional working capacity with an applicable low‐temperature heat source for desorption step.

**Figure 4:**
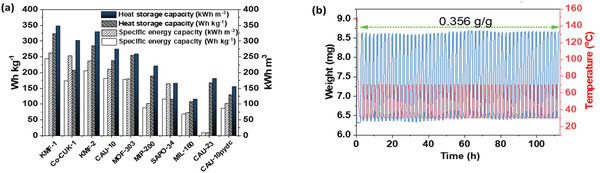
a) Specific energy capacities and heat‐storage capacities for KMF‐2 and benchmark adsorbents. Boundary conditions: heats transferred from the evaporator in one cooling cycle at *T*_ev_ = 5 °C, *T*_ads_ = 30 °C, *T_con_* = 30 °C, and *T*_des_ = 70 °C and heat‐storage capacity at *T*_ev_ = 10 °C, *T_ads_* = 30 ° C, *T_con_* = 30 °C, and *T_des_* = 70 °C. b) TGA profile exhibited by KMF‐2 for 50 water adsorption/desorption cycles. Test conditions: adsorption at 30 °C in humid N_2_ gas (*RH* = 35%) and desorption at 70 °C under N_2_ with low humidity (*RH* = 4.8%). Prior to the multiple cycle experiment, the first cycle was performed under a dry N_2_ gas flow (100 mL min^−1^) after sample dehydration at 150 °C for 1 h.

For a comprehensive assessment of the material performance in AHT application, two additional indicators, i.e., the specific energy capacity (*Q_ev_*) and the heat‐storage capacity (*Q_stored_*), were also considered.^[^ ^25^ ^]^ The former illustrates the cooling power of the material in AC use while the latter is important for daily or seasonal energy storage. Therefore, these parameters were determined for KMF‐2 as well as for the other benchmark adsorbents as shown in **Figure** **4**a and Table S8 (Supporting Information). The temperature boundaries of typical AC conditions were applied to calculate the specific energy (*T*_ev_ = 5 °C, *T*_ads_ = 30 °C, and *T*_des_ = 70 °C). The volumetric *Q_ev_* for KMF‐2 (237 kWh m^−3^) was superior to benchmarks except for KMF‐1 (263 kWh m^−3^) and Co‐CUK‐1 (253 kWh m^−3^). The *Q_stored_* value for KMF‐2 (329 kWh m^−3^) at *T_ev_* = 10 °C, *T_con_* = 30 °C, and *T_des_* = 70 °C, is even higher than that for Co‐CUK‐1 (302 kWh m^−3^) and only slightly lower than KMF‐1 (348 kWh m^−3^). From in‐depth thermodynamic evaluation of KMF‐2 in AHT application, its performance was proven to be enhanced compared with that of CAU‐10H and CAU‐10pydc. CAU‐10pydc showed very low values of *Q_ev_* (102 kWh m^−3^) and *Q_stored_* (154 kWh m^−3^) because its strong hydrophilicity requires high regeneration temperature (<80 °C).^[^ ^32^ ^]^ For CAU‐10H, its values of *Q_ev_* (210.7 kWh m^−3^) and *Q_stored_* (273.7 kWh m^−3^) are also lower than those for KMF‐2 (237 kWh m^−3^ for *Q_ev_* and 329.1 kWh m^−3^ for *Q*_stored_) because of its lower Δ*W* than that for KMF‐2 at given boundary condition. Furthermore, KMF‐2 outperforms most of the MOF benchmarks except for KMF‐1 and Co‐CUK‐1. However, the materials cost and scalable production should be considered for real implementation of industrial use (Tables S9 and S10, Supporting Information). KMF‐1 is limited by the high cost of the organic linker despite its high space‐time to yield under reflux conditions. In case of Co‐CUK‐1, low reaction yield (<33%) and harsh hydrothermal conditions (≈200 °C) are a drawback. Therefore, the slightly lower performance of KMF‐2 compared to KMF‐1 and Co‐CUK‐1 is largely compensated by its rather low material cost (9 times cheaper than Co‐CUK‐1 and 800 times cheaper than KMF‐1) and its facile scalable and eco‐friendly hydrothermal condition.

The long‐term stability of KMF‐2 was further evaluated with a multi‐cyclic water adsorption/desorption test under N_2_ flow consisting of water vapor through the adsorption chamber containing KMF‐2 with *RH* swing between 3.1% and 35% (**Figure** 4b). As a result, KMF‐2 was confirmed to exhibit high stability without loss of initial adsorption capacity of 0.356 g_H2O_ g_MOF_^−1^ up to 50 cycles.

### 2.4 Molecular Simulations

Grand‐Canonical Monte Carlo (GCMC) simulations were performed to shed light on the molecular adsorption mechanism at the origin of the water adsorption performance of KMF‐2. **Figure** 2c reports the comparison between the experimental and the GCMC simulated adsorption isotherms at 30 °C considering the most energetically stable MOF model described in Figure S16 (Supporting Information). Notably, the experimental step at *P*/*P*° = 0.1 is very well reproduced by our calculations and the saturation adsorption uptake is only slightly overestimated (exp: 0.37 g_H2O_ g_MOF_^−1^ and simulation: 0.34 g_H2O_ g_MOF_^−1^). The S‐shaped adsorption profile at the initial stage of adsorption is consistent with a simulated water adsorption enthalpy of about −40 kJ mol^−1^ very similar to the heat of evaporation for water (40.7 kJ mol^−1^). The simulated adsorption enthalpy alongside the water loading (Figure S17, Supporting Information) was found to be on average about −55 kJ mol^−1^ in line with the experimental isosteric enthalpy of adsorption described above. To gain more insight into the water‐MOF interactions, we plotted the most representative radial distribution functions for the water/MOF pair, i.e., between the oxygen atom (Ow) of water and the oxygen atom (Oh) of µ‐OH, the nitrogen (N) atom of the PYDC linker and the oxygen atom of the carboxylate group of the MOF (Figures S18–S21, Supporting Information). This analysis revealed that at the initial stage of adsorption, H_2_O interacts mostly with the oxygen atom of the carboxylate group of KMF‐2 (Figure S19, Supporting Information) while the probability for H_2_O to interact with µ‐OH is rather low as shown by the relatively low intensity of the first peak for the RDF of the Ow‐O (µ‐OH) pair situated at 2.58 Å (Figure S18, Supporting Information) characteristic of a strong hydrogen bond between the water and these hydroxyl groups of the MOF (Figure S23a, Supporting Information). This adsorption behavior significantly differs from the one we previously found for CAU‐10pydc where the water molecules are interacting strongly with the oxygen atom (Oh) of µ‐OH (see Figure S21, Supporting Information, the associated RDF plotted for both MOFs at the initial stage of adsorption).^[^ ^32^ ^]^ Figure S22 (Supporting Information) illustrates this distinct adsorption behavior with the µ‐OH groups being much less accessible to H_2_O in the case of KMF‐2 in line with its lower hydrophilicity as seen in the water adsorption isotherms reported in Figure 2.

**Figure 5:**
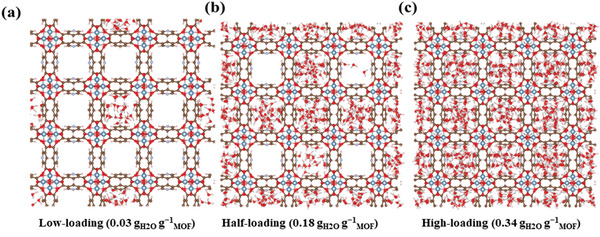
Illustrative GCMC snapshots of the water filling at different adsorption loading in KMF‐2: low, half, and high loading. The gray lines indicate the H‐bonds between water molecules and between water and MOF.

This preferential sitting of the water molecules illustrated in **Figure** **5**a and Figures S23 serves as anchoring sites to host additional water molecules forming a hydrogen bond network with an Ow‐Ow separating distance of 2.75 Å (Figure S24, Supporting Information). The channels are thus filled up one by one (**Figure** 5b) until we reach full saturation (**Figure** 5c). At intermediate and high‐water loading, the Ow/O (µ‐OH) and Ow/O‐carboxylate IPA interactions gradually increase while an additional interaction occurs between the oxygen atom of water and the nitrogen atom of the PYDC linker (Figures S18–S21, Supporting Information). A strong H‐bond network of water adsorbed along *c*‐direction is then achieved when the pore is fully saturated (Figure S25, Supporting Information) with a number of hydrogen bonds per water of 2.9 being similar to the value previously reported for KMF‐1.^[^ ^25^ ^]^

## 3 Conclusion

A new series of mixed‐linker Al MOFs was discovered via a green synthetic procedure implying the use of low‐cost linkers with different hydrophilicity, IPA, and PYDC. The hydrophilicity of the designed materials was shown to be easily tuned through variation of the linker ratio. This enables systematic control of the water adsorption uptake step *P*/*P*° in the water adsorption isotherm. The potential of the Al‐MOF characterized by 50:50 PYDC/IPA ratio, denoted as KMF‐2, revealed a high water uptake of 0.33 g_H2O_ g^–1^_MOF_ at 30 °C under low relative pressure (*P*/*P*° < 0.18). This MOF was shown to display an exceptional level of performance for both AC and AHP applications with not only high COP values for cooling (COP_C_ = 0.75) and heating (COP_H_ = 1.67) but also remarkable specific energy capacity (*Q*_ev_) of 236.7 kWh m^−3^ and energy storage capacity (*Q*_stored_) of 329.1 kWh m^−3^ at *T*_des_ = 70 °C. Multi‐cyclic water adsorption–desorption experiments with up to 50 cycles demonstrated the facile regeneration of this novel MOF with low‐temperature heat source, excellent long‐term stability along with high working capacity (0.350 g_H2O_ g^−1^_MOF_). Moreover, this MOF was shown to exhibit outstanding stability under hydrothermal and acid‐base conditions. Therefore, KMF‐2 offers great potential as a multi‐purpose commercial water adsorbent for both AC and AHP devices thanks to its many advantages such as green synthesis, scalable production, low material cost, and exceptionally high stability.

## 4 Experimental Section

### Synthesis of Material

CAU‐10‐H was synthesized referring to the literature.^[^ ^20^ ^]^ Mixed‐linker Al−*x*IPA‐(100−*x*)PYDC (*x*: feeding ratio of IPA) samples were synthesized through either reflux or hydrothermal reactions. Mixture of various ratios of isophthalic acid and 3,5‐pyridinedicarboxilic acid (total amount: 40 mmol) and NaOH (120 mmol, 4.80 g) was dissolved into 108 mL of deionized water (refer to Table S1, Supporting Information for more detail). The solution was then added to the solution of AlCl_3_∙6H_2_O (40 mmol, 9.66 g) in 108 mL of deinonized water, dropwise. The mixture was then heated at 120 °C for 12 h under either reflux or hydrothermal condition. The white product was filtered, washed with deionized water and ethanol, and dried at 100 °C overnight.

Characterization of materials, thermodynamic calculations, evaluation of activation energy for desorption, and molecular simulations are described in the Supporting Information.

## Supporting information

Supporting Information

## Data Availability

The data that support the findings of this study are available from the corresponding author upon reasonable request.
