# Supplementary material for: Tuning Hydrophilicity of Aluminum MOFs by a Mixed‐Linker Strategy for Enhanced Performance in Water Adsorption‐Driven Heat Allocation Application
Source: Adv Sci (Weinh). 2023 May 13;10(21):2301311. doi: 10.1002/advs.202301311 (PMC10375118; doi:10.1002/advs.202301311)
Supplement: Supplementary file 1 — Supporting Information [file ADVS-10-2301311-s001.pdf]

ADVANCED SCIENCE
Open Access

Supporting Information

for *Adv. Sci.*, DOI 10.1002/advs.202301311

Tuning Hydrophilicity of Aluminum MOFs by a Mixed-Linker Strategy for Enhanced Performance in Water Adsorption-Driven Heat Allocation Application

Bao N. Truong, Daiane D. Borges, Jaedeuk Park, Ji Sun Lee, Donghui Jo, Jong-San Chang, Sung June Cho, Guillaume Maurin*, Kyung Ho Cho* and U-Hwang Lee*

Supporting Information

Tuning Hydrophilicity of Aluminum MOFs by a Mixed-Linker Strategy for Enhanced Performance in Water Adsorption-Driven Heat Allocation Application

Bao N. Truong, Daiane D. Borges, Jaedeuk Park, Ji Sun Lee, Donghui Jo, Jong-San Chang, Sung June Cho, Guillaume Maurin,* Kyung Ho Cho,* and U-Hwang Lee*

B. N. Truong, J. Park, J. S. Lee, D. H. Jo, U. H. Lee, J. S. Chang, K. H. Cho
Research Group for Nanocatalyst (RGN) and Chemical & Process Technology Division
Korea Research Institute of Chemical Technology (KRICT)
Gajeong-Ro 141, Yuseong, Daejeon 34114, Republic of Korea
E-mail: khcho@krict.re.kr

B. N. Truong, U. H. Lee
Department of Advanced Materials and Chemical Engineering
University of Science and Technology (UST)
Gajeong-Ro 217, Yuseong, Daejeon, 34113, Republic of Korea

J. S. Chang
Department of Chemistry
Sungkyunkwan University
Seobu-Ro 2066, Jangan-gu, Suwon 16419, Republic of Korea

D. D. Borges, G. Maurin
ICGM
Univ. Montpellier, CNRS, ENSCM
Montpellier 34095, France
E-mail: guillaume.maurin1@umontpellier.fr

D. D. Borges
Institute of Physics
Federal University of Uberlândia
Uberlândia-MG 38408-100, Brazil

S. J. Cho
Department of Chemical Engineering
Chonnam National University
Yongbong-Ro 77, Buk-gu, Gwangju 61186, Republic of Korea
Email: sjcho@chonnam.ac.kr

Characterization of materials. PXRD patterns of the samples were obtained using a X-ray diffractometer (Malvern Panalytical Aeris XRD) equipped with Ni-filtered CuK_{α}-radiation (40 kV, 15 mA, λ = 1.5419 Å) and a PIXcel1D-Medipix3 detector. High-temperature PXRD data were measured by Rigaku Ultima IVO (3kW) model equipped with a high temperature cell after kept for 15 min at measuring temperature under He flow. Scanning electron microscopy (SEM) images were collected by Tescan Mira 3 LMU FEG at 10 kV acceleration voltage after coating the samples with Pt in Quorum Q 150T ES equipment. ^{1}H NMR was performed on a Bruker Avance-700 FT-NMR Spectrometer after dissolving 10 mg of samples into 1 mL of 10 wt% $NaOD/D_2O$ solution. The quantitative C, H, N and Al elemental analysis of the samples was determined by Thermo Scientific FLASH 2000 series elemental analyzer and Thermo Scientific iCAP 7400 duo Inductively Coupled Plasma-Atomic Emission Spectrometer (ICP-AES). To analyze the porosity of samples, the activation of samples was carried out at 150 °C for 12 h under vacuum (~10^{-5} Torr). N_2 isotherms were measured by a volumetric sorption analyzer (Micromeritics 3flex) at liquid N_2 temperature (−195.8 °C). The BET surface area (S_{BET}) was calculated using the Brunauer–Emmett–Teller equation from adsorption isotherm curve, and the total pore volume (V_P) was measured by a single point method at P/P° = 0.95. Furthermore, the micropore surface area (S_{micro}) and the micropore volume ($V_{P,micro}$) were determined by the t-plot method. To obtain the pore size distribution (PSD) of the micropore of samples, Ar isotherm was acquired at liquid Ar temperature (−185.8 °C) using ASAP 2020 equipment. The PSD were fitted by its Ar adsorption isotherm curve with the cylindrical model of Horvath–Kawazoe equation with Ar–oxide model. The thermogravimetric curve (TG) was obtained with the Scinco TGA i-1000 model under 30 cm^3 min^{-1} N_2 with a 5 °C min^{-1} ramping rate after exposure to water vapor at 80% relative humidity in a chamber controlled by the NH_4SO_4 salt solution.

Water sorption measurements. Water sorption isotherms of samples were measured by an intelligent gravimetric analyzer (IGA, Hiden Analytical Ltd.). The IGA was automatically operated to finely control the water vapor pressure (RH of 1–95%) and temperature (20–100 °C). Before measuring the water adsorption experiments, the samples were degassed at 150 °C for 6 h under high vacuum (< 10^{-6} Torr). Multiple cycles of water adsorption-desorption were carried out using a thermogravimetric analyzer (TGA, DT Q600, TA Instruments, Universal V4.5A) connected with a humidity generator. The humidity of gas stream was controlled using two mass flow meter, and a humidified nitrogen gas flow was passed through a thermogravimetric chamber. During the water

adsorption–desorption cyclic measurement, the weight of dehydrated sample was determined by first adsorption cycle after activated at 150 °C for 1 h. After then, the adsorption profiles were measured at 30 °C in humid nitrogen at 35% of RH, while desorption data were collected at 70 °C in 4.8% of *RH* nitrogen flow at a cycle time of 1.5 h.

Water desorption kinetic. The desorption experiments of the adsorbents were conducted by the same TGA instrument (DT Q600, TA instruments, Universal V4.5A). Before the experiments, all samples were allowed to adsorb water to saturation under humid flow gas of relative humidity of 60% for 90 minutes. Then, the samples were heated from 30 °C to 150 °C under various heating rates of 4, 6, 8, 10, and 12 ºC min^{-1} under humid flow gas mentioned above. The sample weight for the measurement was about 10 mg.

Molecular simulations. The initial experimental structural model of the hydrated KMF-2 was geometry optimized by Density functional theory (DFT) calculations maintaining fixed the cell parameters. These DFT calculations were carried out within the Gaussian Plane Waves method as implemented in CP2K package within Generalized Gradient Approximation (GGA) with the Perdew-Burke-Ernzerhof (PBE) exchange functional.[1] The energy cutoff for the plane waves is set at 600 Ry. Goedecker, Teter, and Hutter (GTH) pseudo-potentials approximation[2] and double zeta basis sets (DZVP)[3] were employed while DFT-D3 van der Waals dispersion corrections were considered with a cut-off radius of 10 Å.[4] The Density Derived Electrostatic and Chemical (DDEC) net atomic charges[5] were further computed using the DFT optimized electron density as an input (see the cif file of the DFT optimized structure incorporating the charges). The textural properties including theoretical free pore volume, pore limiting diameter and N_2-accessible surface area were computed using a geometric method as implemented in the Zeo++ software package. Grand-Canonical Monte Carlo (GCMC) simulations were performed to predict the water adsorption isotherms at $T_{ads} = 30$ °C. The simulation box was made of 12 conventional unit cells (2×2×3) maintaining the atoms fixed in their initial positions. The interactions between the guest water molecules and the MOF structure were described by a combination of site-to-site LJ contributions and Coulombic terms. The Lennard-Jones (LJ) parameters for all atoms of the MOF framework were taken from the generic UFF forcefield.[6] Following the treatment adopted previously for MIL-160,[7] the hydrogen atoms of both the μ-OH moieties interact with water via only a Coulombic term. The TIP4P/2005 model[8] was used as a microscopic model to represent the water molecule. Short-range dispersion forces were truncated at a cutoff radius of 12 Å while

the cross-term LJ parameters were calculated by means of the Lorentz–Berthelot combination rule. The long-range electrostatic interactions were handled using the Ewald summation technique. For each point of the adsorption isotherm, $4x10^8$ MC cycles were considered to ensure the convergence. In order to gain insight into the configurational distributions of the water in the MOF, additional data were calculated at different pressure including the hydrogen bond networks and the radial distribution functions (RDF) of the intermolecular atomic pairs of the water and the MOF framework as well as water-water.

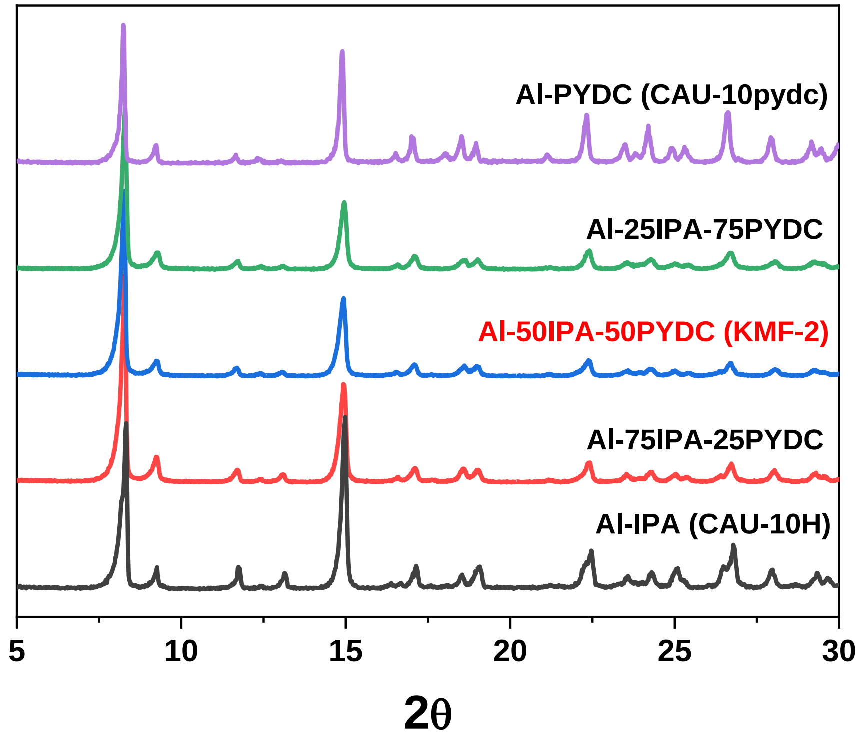

Figure S1. Comparison of PXRD patterns of the Al-xIPA-(100-x)PYDC samples with CAU-10H and CAU-10pydc.

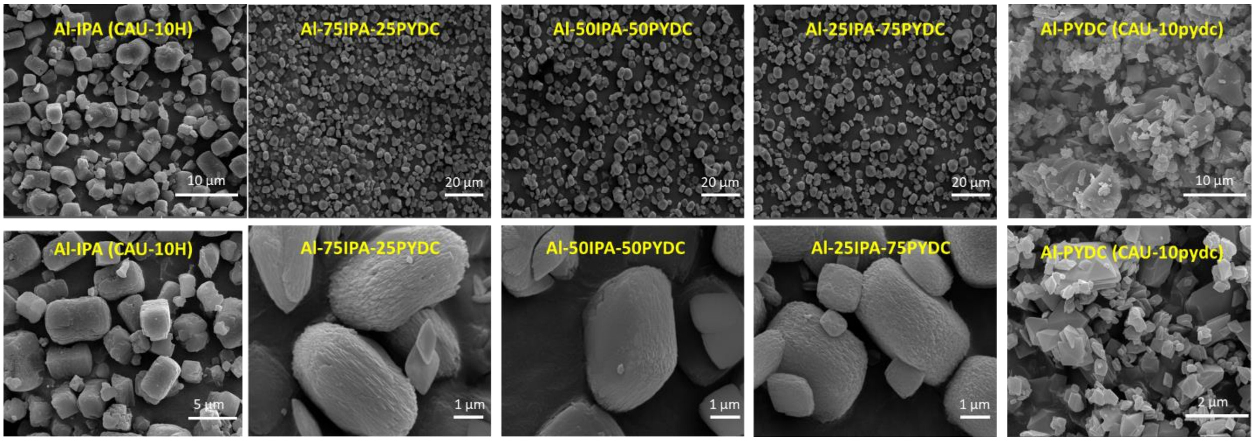

Figure S2. SEM images of the Al-xIPA-(100-x)PYDC samples.

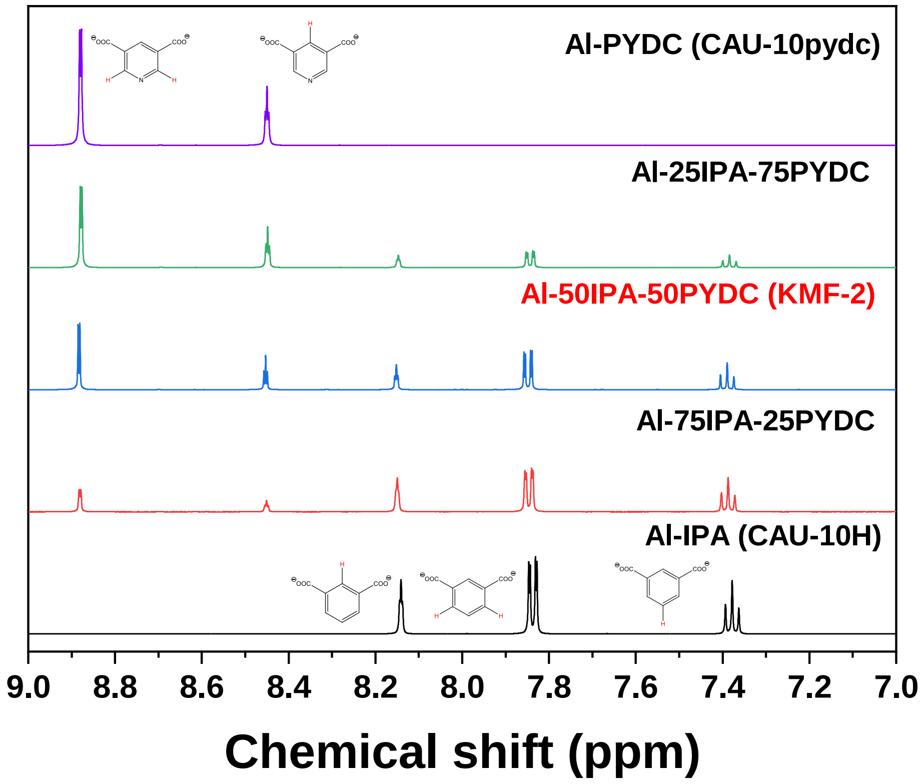

Figure S3. ^{1}H NMR spectrum of Al-xIPA-(100-x)PYDC samples, CAU-10H and CAU-10pydc after digestion in 10% NaOD in D_2O.

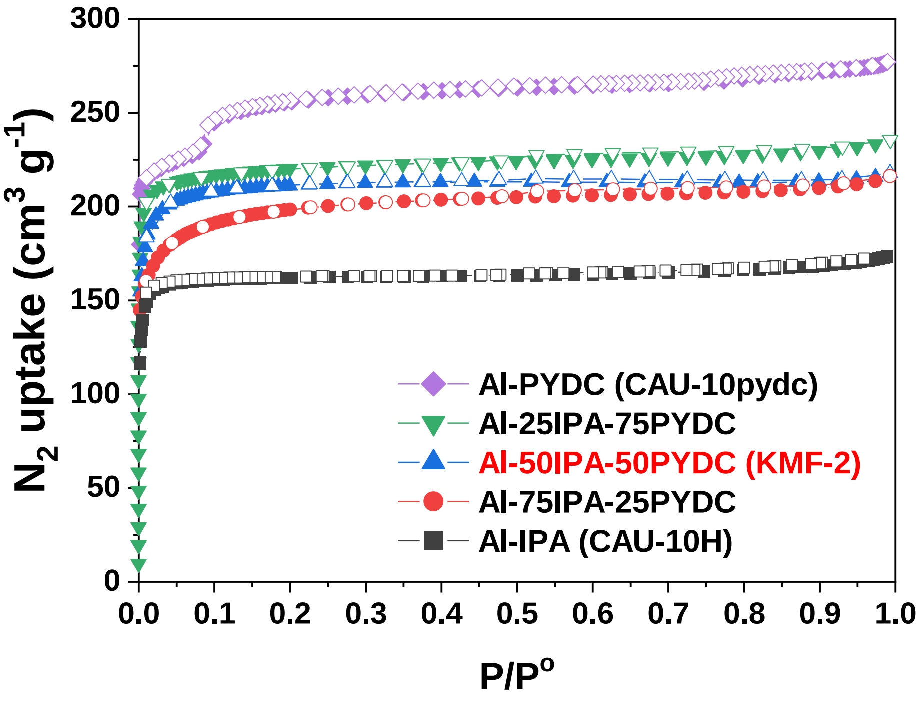

Figure S4. N_2 adsorption isotherms of the Al-xIPA-(100-x)PYDC samples, CAU-10H and CAU-10pydc at −195.8 °C after activation 150 °C for 12 h.

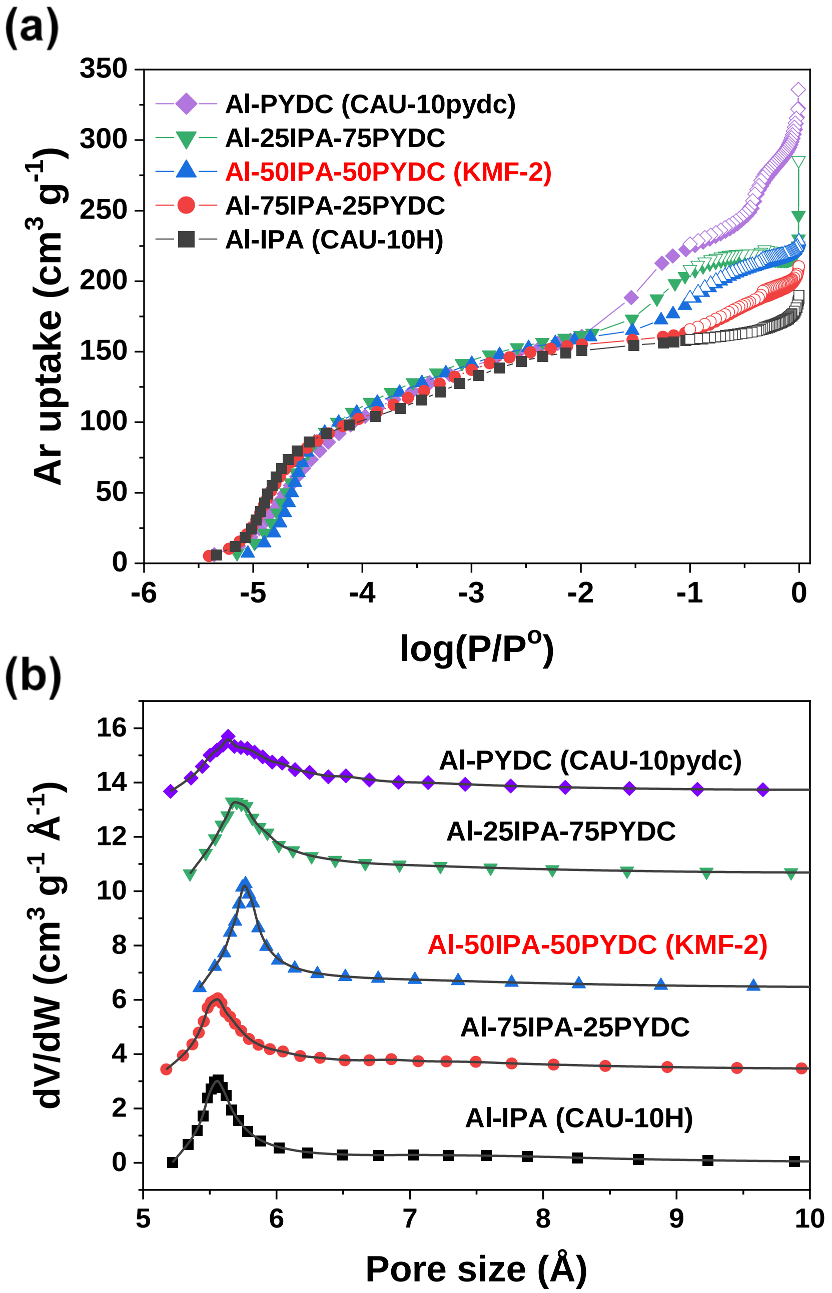

Figure S5. Argon sorption measurement for the Al-xIPA-(100-x)PYDC samples, CAU-10H and CAU-10pydc at −185.8 °C after activation 150 °C for 12 h. **(a)** Argon isotherm plotted with a logarithmic P/P° and **(b)** Pore size distribution determined by Horvath–Kawazoe equation.

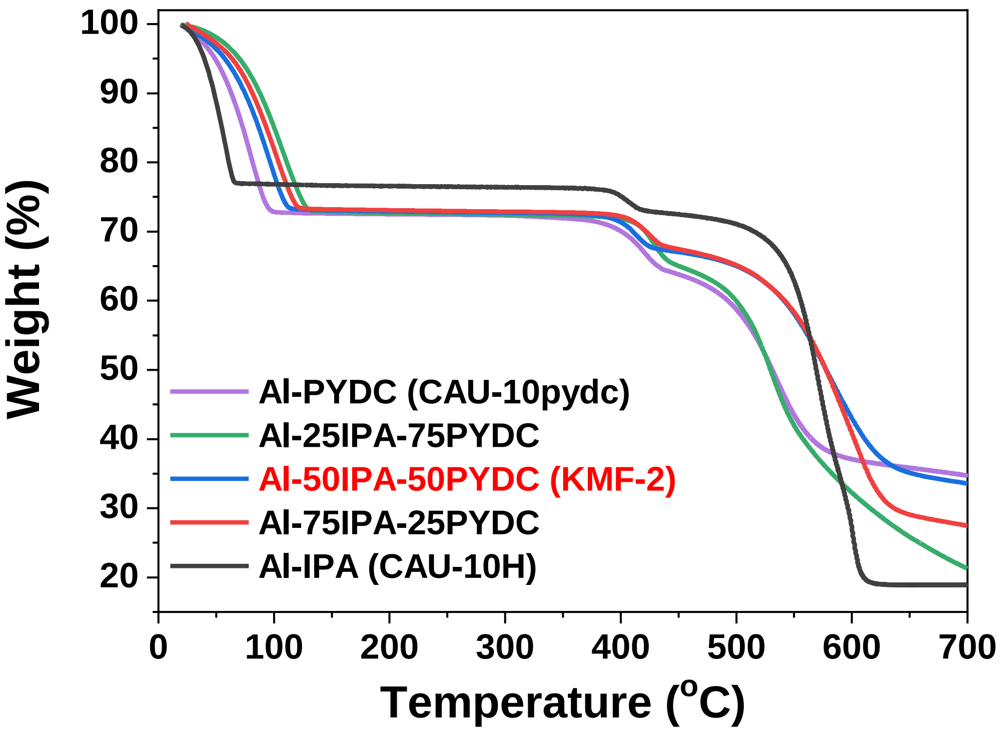

Figure S6. TG curves of Al-xIPA-(100-x)PYDC samples, CAU-10H and CAU-10pydc under 30 cm^3 min^{-1} N_2 flow with 5 °C min^{-1} ramping rate. Before TG analysis, the samples were exposed to ~80% of relative humidity chamber controlled by NH_4SO_4 salt solution.

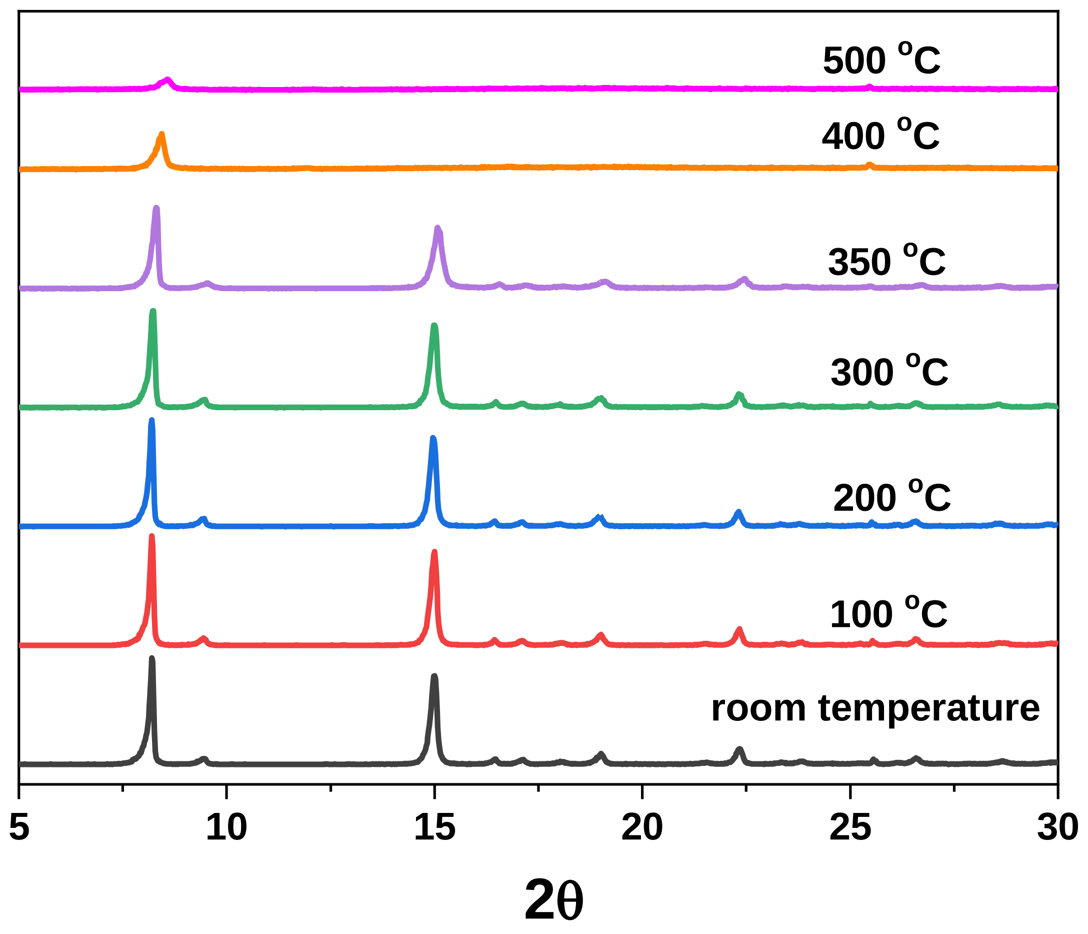

Figure S7. High-temperature PXRD (HT-PXRD) patterns of KMF-2 measured from room temperature to 500 °C.

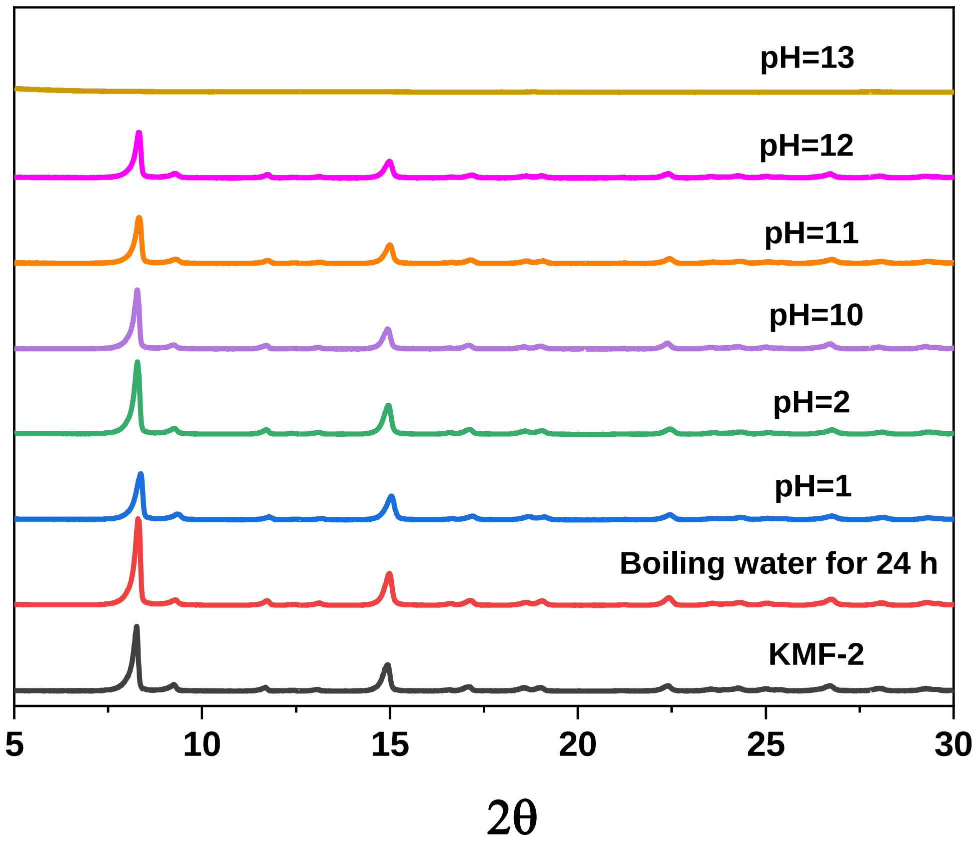

Figure S8. PXRD patterns of KMF-2 after hydrothermal and pH stability test. For hydrothermal test, the sample was immersed in water and heated at 100 °C for 24 h. For the pH test, the sample was soaked into the acidic or basic aqueous solution (pH 1−13) for 24 h at room temperature.

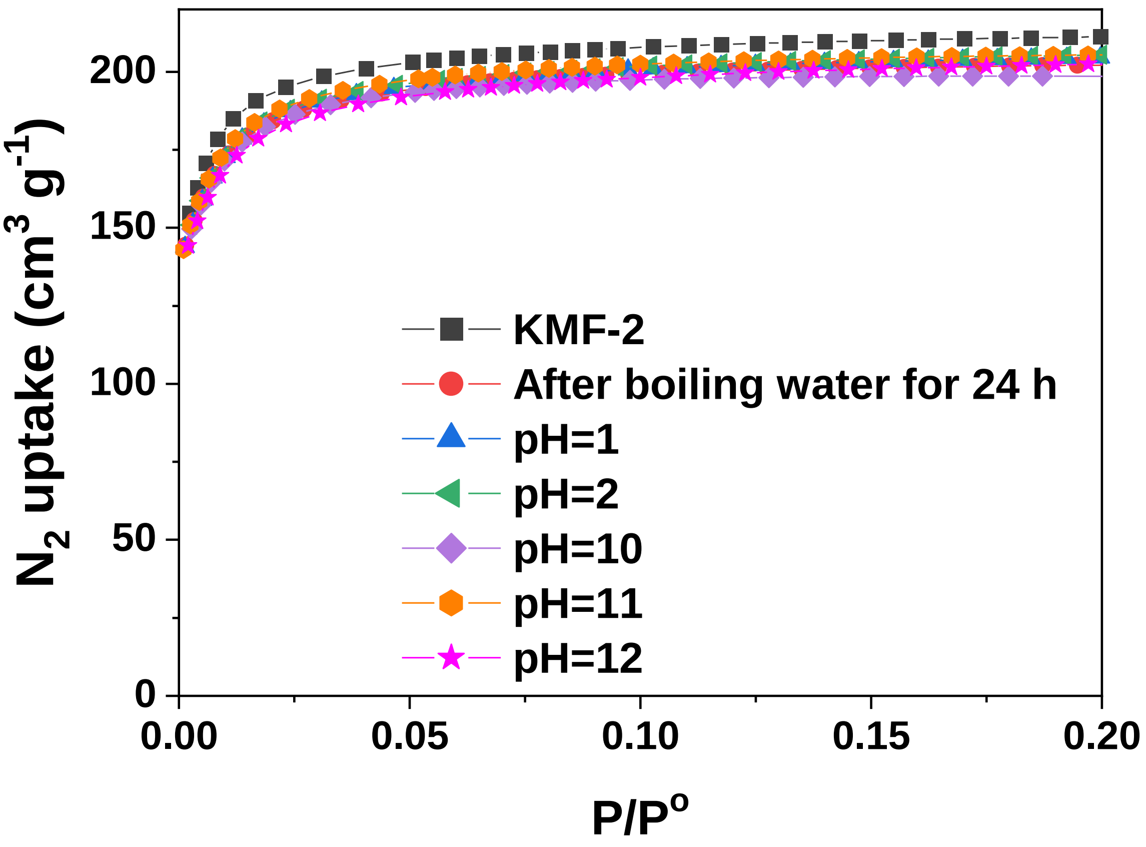

Figure S9 . N_2 adsorption isotherm of KMF-2 after hydrothermal and pH stability test.

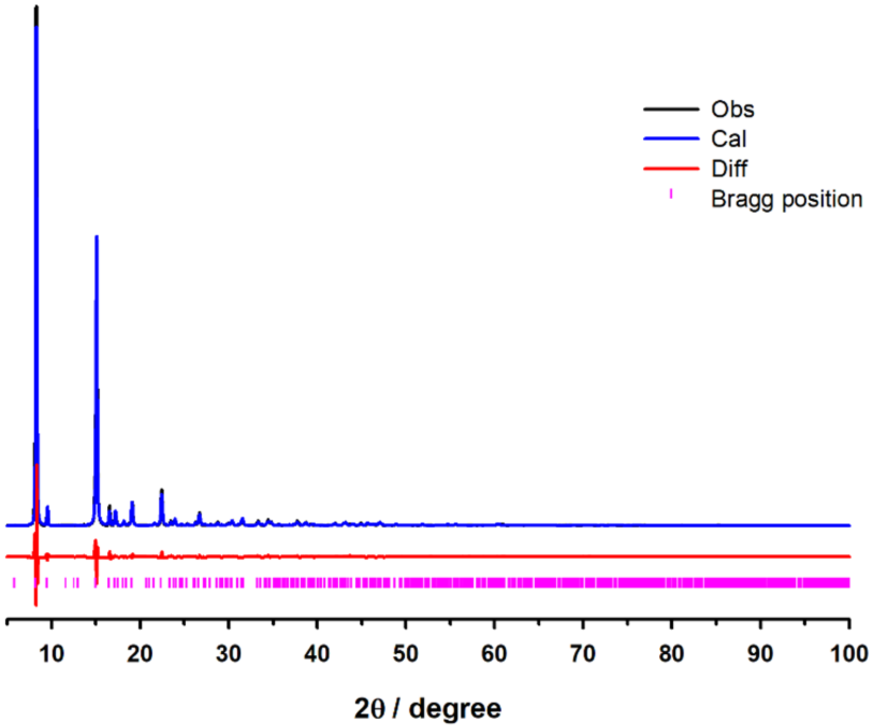

Figure S10. Rietveld refinement of the KMF-2 dehydrated structure models. The experimental data are shown in black, simulation in blue, and difference in red. Allowed Bragg reflections are indicated as magenta ticks below.

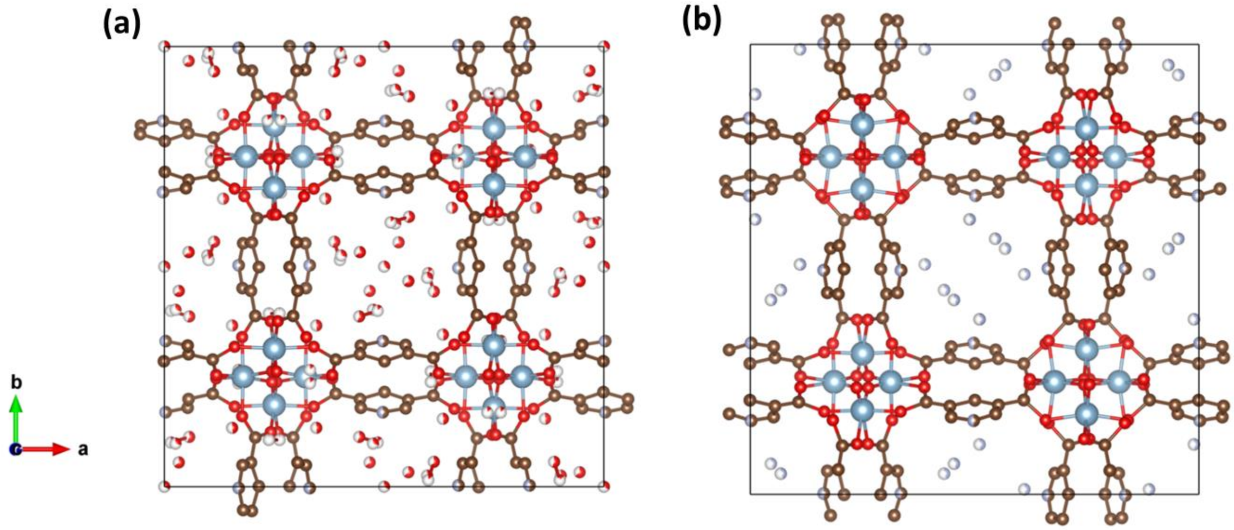

Figure S11 Crystal structure of KMF-2 for (a) hydrated and (b) dehydrated form, respectively.

Determination of the isosteric heat of adsorption

To assess the COP of a working pair, knowledge of the enthalpy of adsorption is of prime importance.[7] As described in more detail in Ref. [7], isosteric heats of adsorption were estimated by the Clausius-Clapeyron equation and compensated by the virial equation.[9] The isosteric enthalpy of adsorption was calculated from the isotherms at multiple temperatures by Eq. (S1):

$$\Delta_{ads}H_w = R\left(\frac{\partial lnp}{\partial(^1/_T)}\right)_w \quad \text{(S1)}$$

where $\Delta_{ads}H_W$ is the isosteric enthalpy of adsorption, R is the universal gas constant, p and T represent temperature and pressure, respectively, and W is the volume of water (liquid) adsorbed per volume of adsorbent (crystalline densities are used for the conversion).[7]

Furthermore, the virial equation was also employed to calculate the isosteric heat of adsorption (Q_{st}) from isotherms measured at different temperatures.[9]

$$\text{Ln}\, p = \ln v + \left(\frac{1}{T}\right) R \sum_{i=1}^{m} a_i v^{i-1} + \sum_{i=1}^{n} b_i v^{i-1} \quad \text{(S2)}$$

where v is quantity adsorbed, and a_i and b_i are empirical parameters. The virial-type equation was applied compared with the Clausius-Clapeyron equation, as shown in **Figure S12**. A set of temperature-independent parameters, which lead to direct evaluation of Q_{st} can be derived by fitting Eq. (S2) continuously using adsorption isotherms obtained at different temperatures.[9]

$$Q_{st} = -R\left(\frac{\partial \ln p}{\partial\left(\frac{1}{T}\right)}\right)_{v_v} = -R \sum_{i=1}^{m} a_i v^{i-1} \quad \text{(S3)}$$

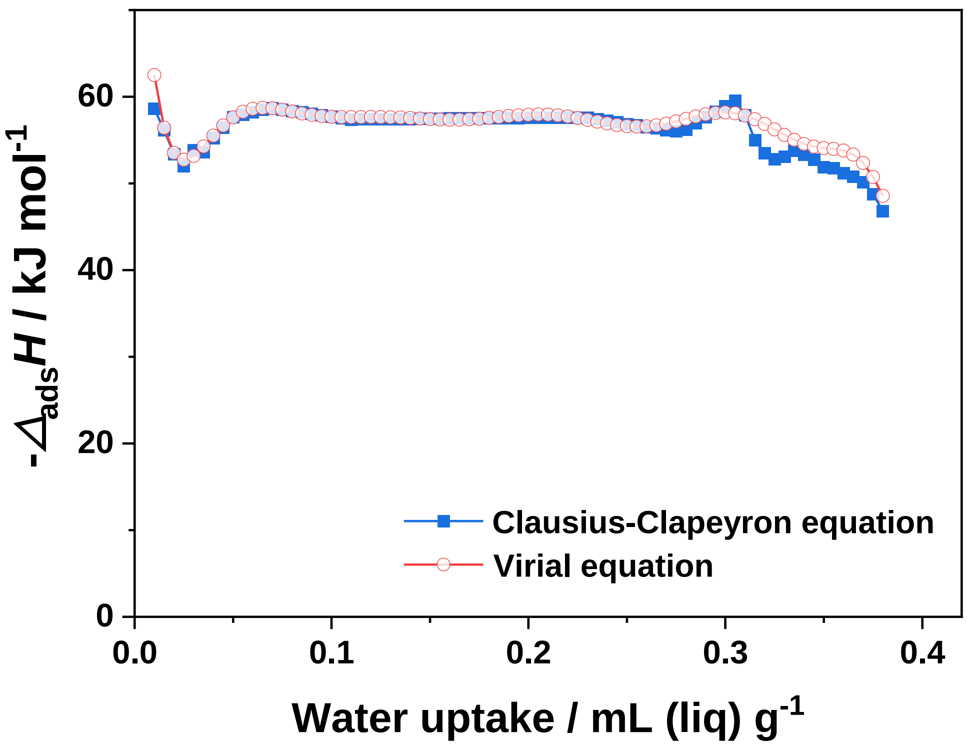

Figure S12. Isosteric heat of adsorption of KMF-2 as a function of water loading determined by both Clausius–Clapeyron and Virial equation.

Evaluation of activation energy for desorption (Ed)

Activation energy of desorption (E_{d}) is derived from Kissinger equation.[10]

$$\frac{d(\ln\frac{\beta}{T_d^2})}{d(\frac{1}{T_d})} = -\frac{E_d}{R} \qquad \text{(S4)}$$

where T_d (K) is temperature at the highest desorption rate recorded through derivative thermogravimetry (DTG) curve, β (K min^{-1}) is heating rate, R is ideal gas constant (J K^{-1} mol^{-1}). Practically, TGA of the saturated adsorbent under various heating rate is performed. Based on DTG curves of the sample, T_d values under various heating rates are collected. Finally, E_d is calculated from slope of linear plot of β/T_d^2 to $1/T_d$.

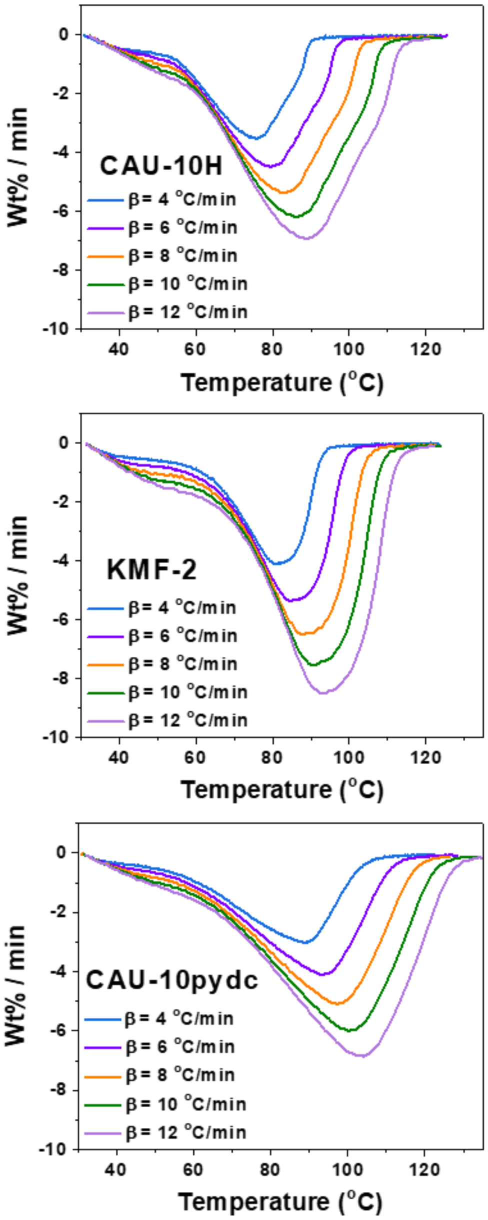

Figure S13. DTG curves of water desorption of CAU-10H, KMF-2, and CAU-10pydc under various heating rates.

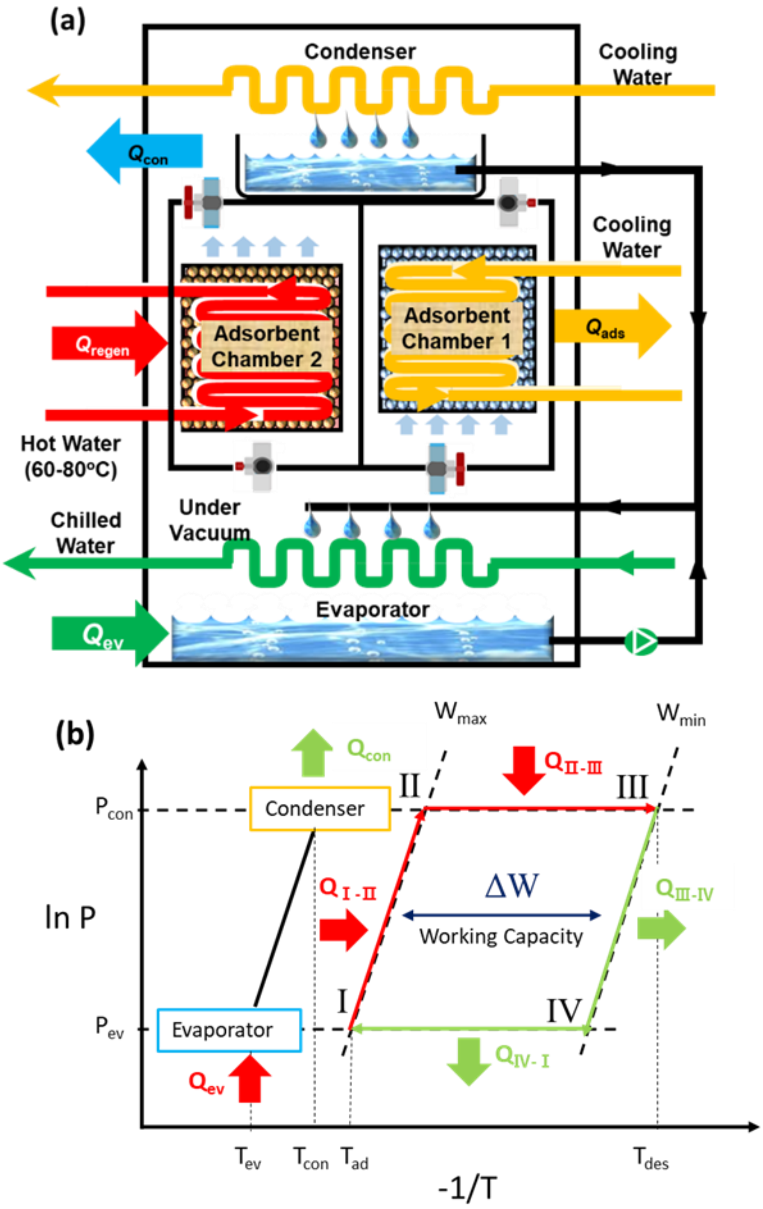

Figure S14. (a) Basic working cycle of AHT devices with adsorption stage (chamber 1) and desorption stage (chamber 2). The releasing heat by the condensation and adsorption is depicted by blue and yellow arrows, respectively, the heat for the desorption by the systems is red arrow, the heat of evaporation is shown as green arrow. **(b)** Thermodynamic cycle of an adsorption heat pump; Arrows point to the flow of energy to or from the heat pump cycle at various temperatures. An adsorption driven heat pump cycle consists of four steps: (I-II) isosteric heating; (II-III) isobaric desorption; (III-IV) isoteric cooling; (IV-I) isobaric adsorption.

Thermodynamic calculations. The thermodynamic evaluation of adsorption cooling and heat pump cycles were calculated using the methodology previously reported by De Lange *et al*.[7] The coefficient of performance (COP) is used to evaluate the energy efficiency of the cooling and heat pump cycle from a thermodynamic perspective. The energy analysis allows the determination of the COP, which is a ratio of useful heating or cooling energy output provided to work required. Calculation details were provided as follows

Detailed procedures of thermodynamic evaluation for AHT application

For calculation procedures, a characteristic curve needs to be constructed to transfer the loading from two dependent variables (*p*, *T*) to one, i.e., the adsorption potential, *A*, which is defined as the molar Gibbs free energy of adsorption with an opposite sign, defined as:

$$A = RTln\left(\frac{P_O(T)}{P}\right) \quad \text{(S5)}$$

where P° is the temperature-dependent vapor pressure of the adsorbate of choice. The amount adsorbed should be expressed as the volume occupied by the adsorbed phase. As the density of the adsorbed phase is often not known, the liquid phase density is used as an approximation:

$$W = \frac{q(p,T)}{\rho_{liq}^{wf}(T)} \Rightarrow W(A) = W_{max}exp\left[-\left(\frac{A}{E}\right)^n\right] \quad \text{(S6)}$$

where *q* is the mass adsorbed, W is the volume liquid adsorbed, and ρ_{liq}^{wf} is the liquid density of the same adsorbate and E is the characteristic energy of adsorption.[11] If temperature invariance is assumed, all measured adsorption data should collapse onto one single “characteristic curve”. An adsorption-driven heat pump cycle can be used for either heating or cooling. As the working pair (sorbent-sorbate) is known, only four distinct temperature levels need to be defined to determine the COP for either application.[7] Notably, the desorption temperature was varied to investigate the required desorption temperature. The COP is defined as the useful energy output divided by the energy required as input. For heating, this becomes:

$$COP_H = \frac{-(Q_{con}+Q_{ads})}{Q_{regen}} \quad \text{(S7)}$$

where Q_{con} is the energy released during condensation, and Q_{ads} is the energy released during adsorption. Both have a negative value, as energy is withdrawn from the adsorption cycle. Q_{regen} is the energy required for regeneration of adsorbent. In this case, a positive quantity as energy is added to the system. For cooling, the coefficient of performance COP_C becomes:

$$COP_c = \frac{Q_{ev}}{Q_{regen}} \quad \text{(S8)}$$

where Q_{ev} is the energy withdrawn by the evaporator. Notably, the COP_H should have a value between 1 and 2, and the COP_C is per definition not larger than unity. The specifics on how to exactly calculate these energetic contributions are explained in detail elsewhere.[7] Finally, it must be noted that the specific heat capacity is assumed to be 1 J/(g · K), which is an average value for a variety of MOF materials. In fact, the actual value of this quantity has a negligible effect on calculated COP values.

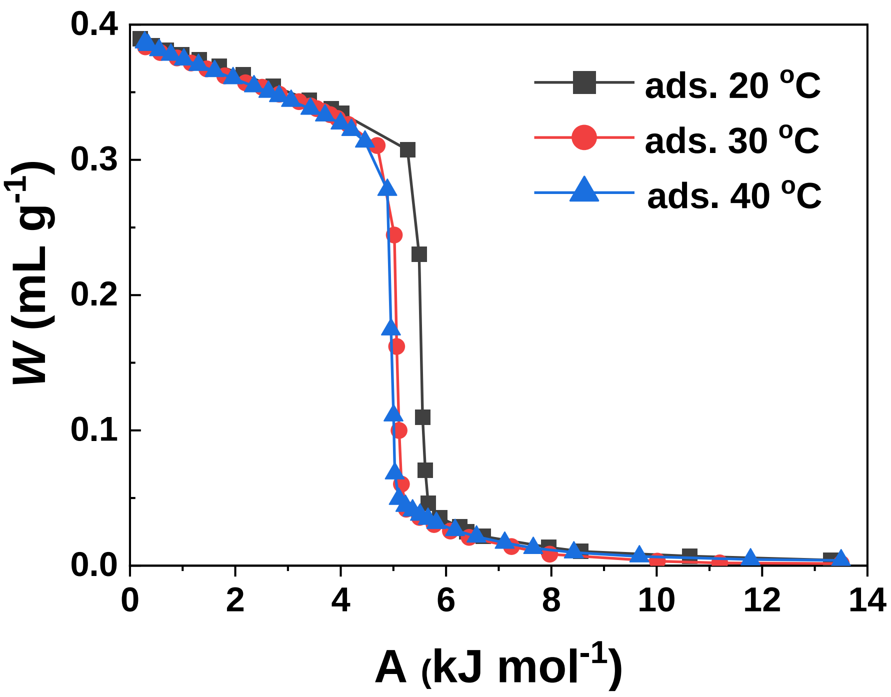

Figure S15. Characteristic curves for KMF-2 as a function of the adsorption potential (A) according to T_{ads} = 20–40 °C determined by Eq. (S6).

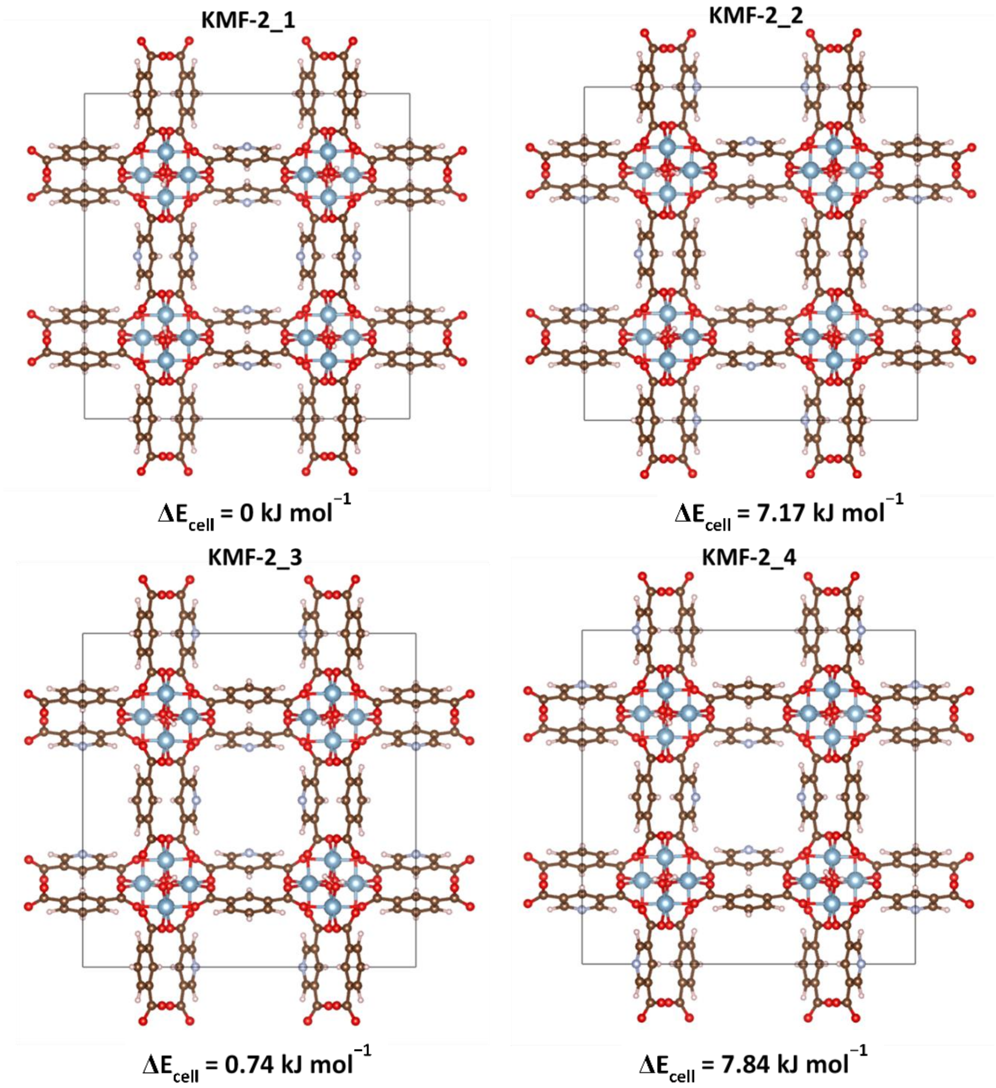

Figure S16. DFT-optimized structures for the 4 possible KMF-2 models corresponding to distinct distribution of PYDC in the unit cell. The electronic energy is reported for the 4 structures with the most stable ones taken as energy reference.

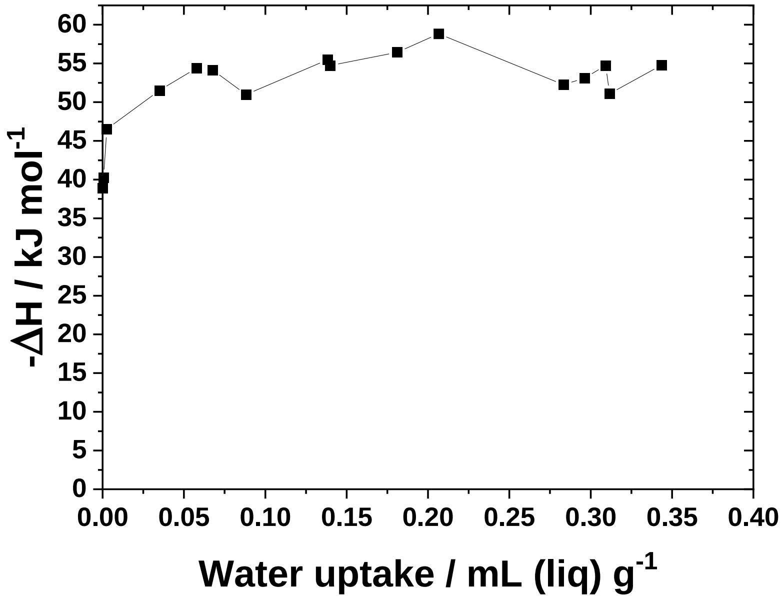

Figure S17. GCMC simulated water adsorption enthalpy as a function of the water uptake for KMF-2 at 30 °C.

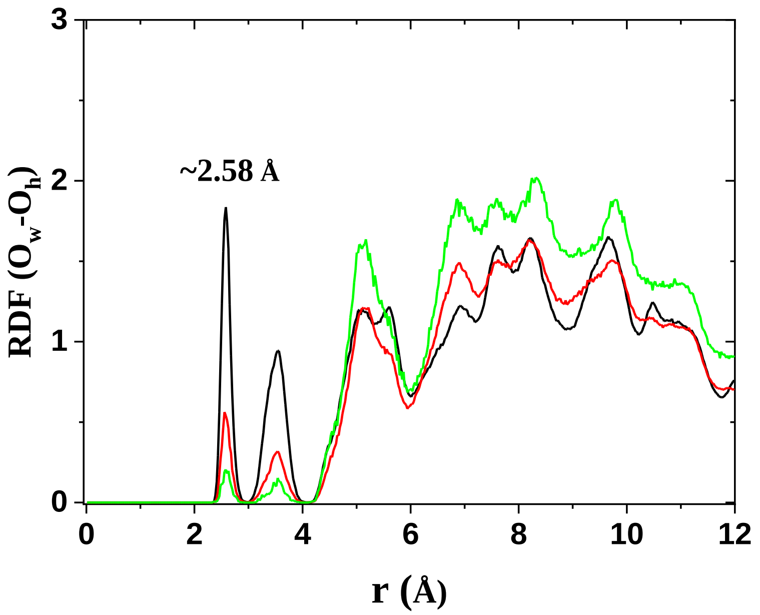

Figure S18. Radial Distribution functions calculated for KMF-2 between the Oxygen atom of water and the Oxygen atom of the μ-OH KMF-2 framework at low loading (green): 0.03 g_{H2O} g_{MOF}^{-1}, half saturation loading (red): 0.18 g_{H2O} g_{MOF}^{-1} and saturation loading (black): 0.34 g_{H2O} g_{MOF}^{-1}.

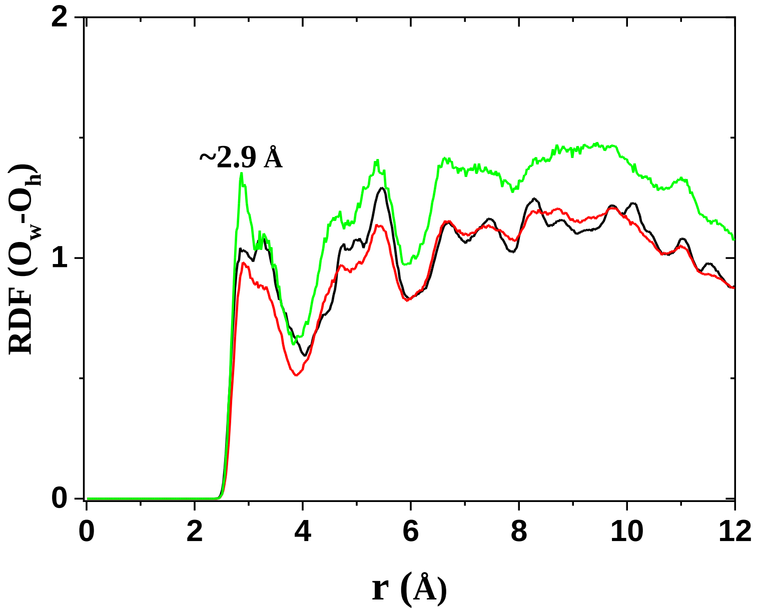

Figure S19 . Radial Distribution function calculated for KMF-2 between the Oxygen atom of water and oxygen atom of the carboxylate group of IPA and PYDC linker at low loading (green): 0.03 g_{H2O} g_{MOF}^{-1}.

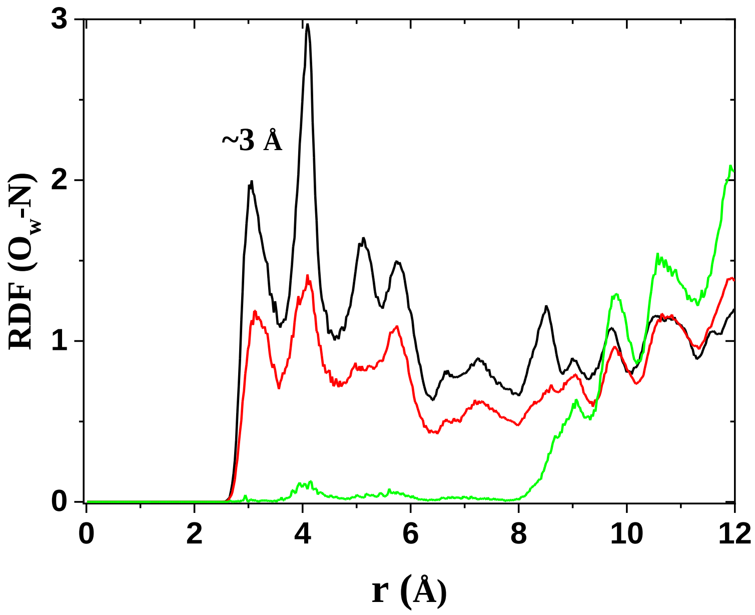

Figure S20. Radial Distribution function calculated for KMF-2 between the Oxygen atom of water and Nitrogen of PYDC linker at low loading (green): 0.03 g_{H2O} g_{MOF}^{-1}, half saturation loading (red): 0.18 g_{H2O} g_{MOF}^{-1}, and saturation loading (black): 0.34 g_{H2O} g_{MOF}^{-1}.

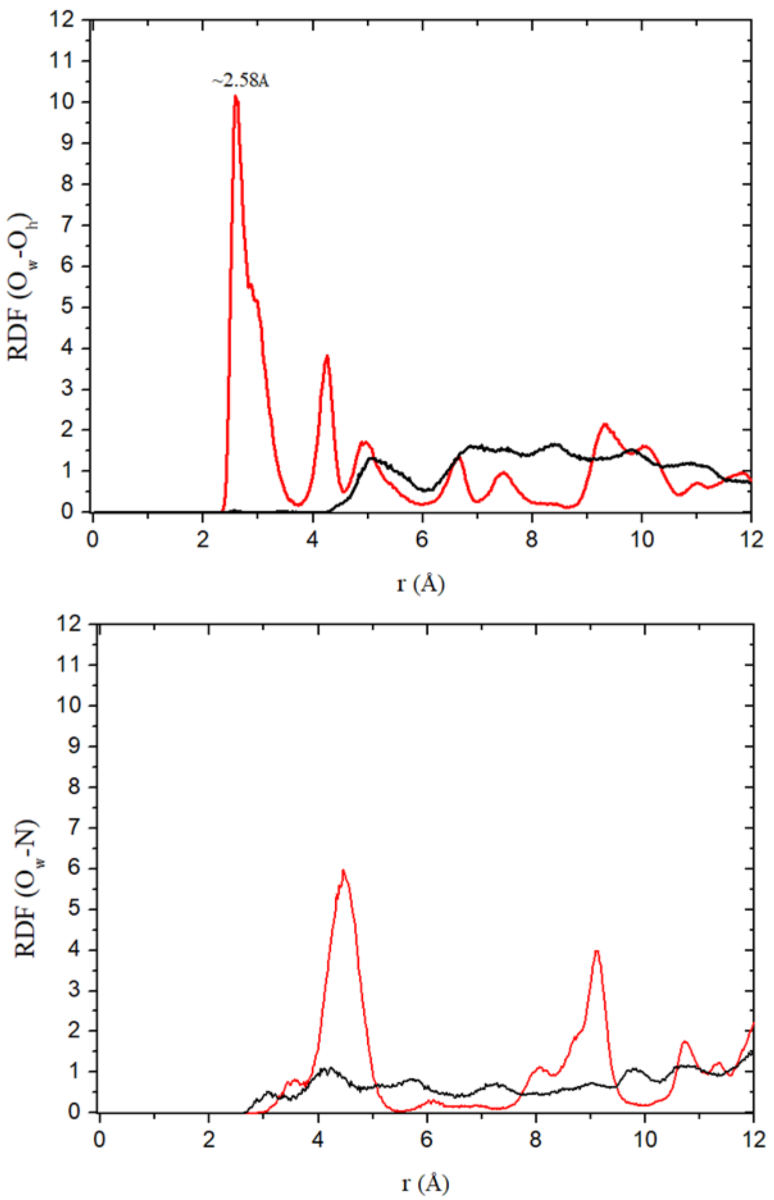

Figure S21. Comparison between the RDFs calculated between Oxygen atoms of water and the Oxygen atom of the μ-OH (top side) and the Nitrogen of PYDC linker (bottom side) for KMF-2 (black) and CAU-10pydc (red) at low loading, (e.g. 0.03 $g_{H2O}\ g_{MOF}^{-1}$).

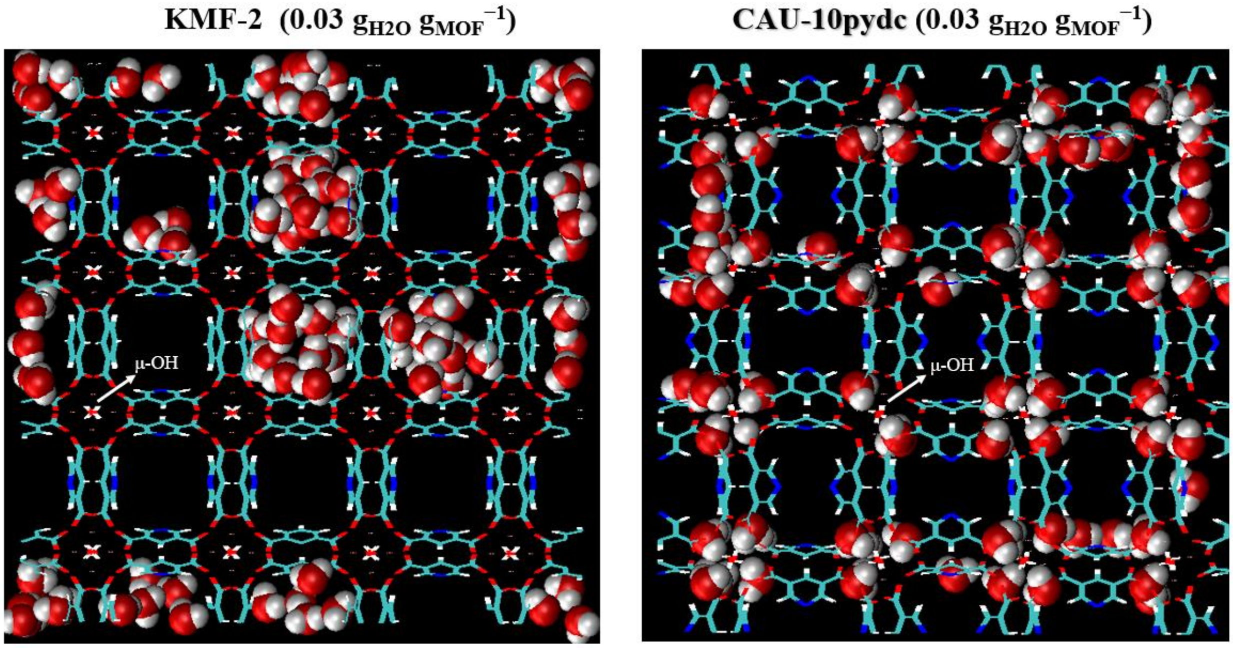

Figure S22. Illustrative snapshots for the water adsorption at low loading in KMF-2 (left) and CAU-10pydc (right).

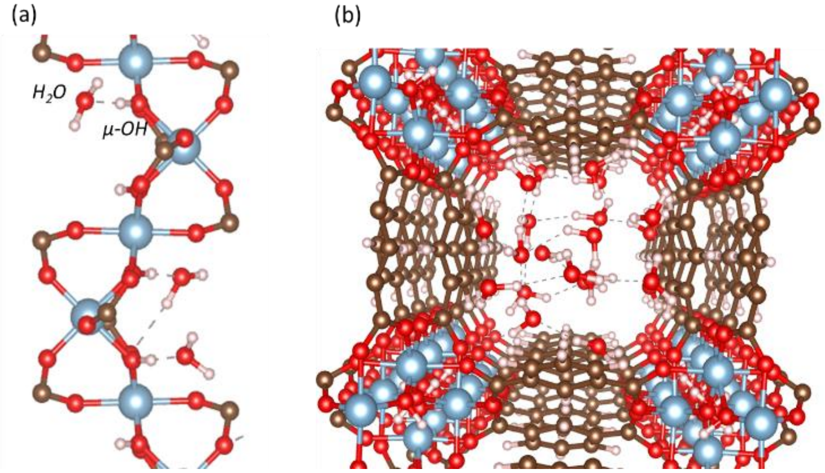

Figure S23. GCMC simulated water organization in KMF-2 at the initial stage of adsorption (a) H-bond formation between Oxygen of water and the μ-OH function of the MOF (b) Subsequent water adsorption filling in 1 channel of the MOF. The dash gray lines indicate the H-bond.

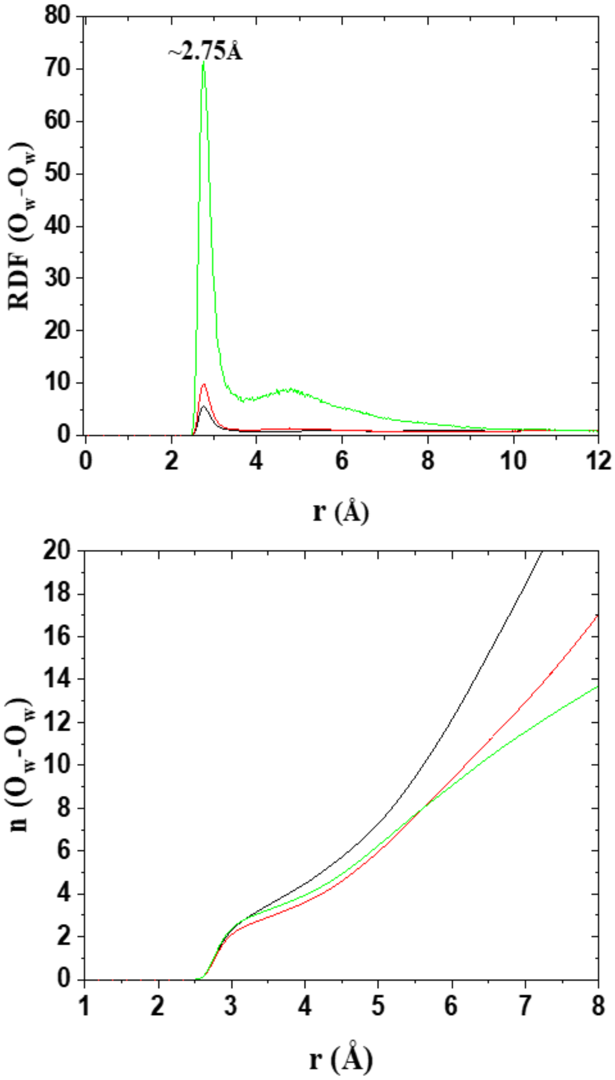

Figure S24 . Radial Distribution function calculated between Oxygen atoms of water themselves (top side) and the coordination number (bottom side) at low loading (green): 0.03 g_{H2O} g_{MOF}^{-1}, half saturation loading (red): 0.18 g_{H2O} g_{MOF}^{-1} and saturation loading (black): 0.34 g_{H2O} g_{MOF}^{-1}.

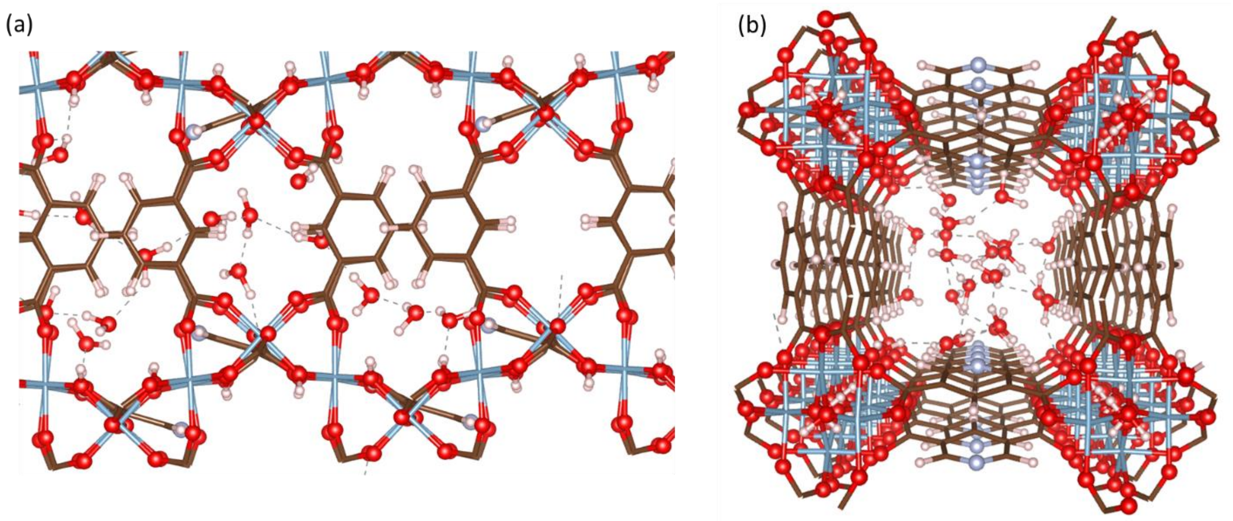

Figure S25. Snapshots showing the H-bond network formed inside the pore. The dash gray lines indicate the H-bonds.

Table S1. Synthetic condition of the materials.

Compound	Ratio of Al^{3+}	Ratio of IPA	Ratio of PYDC	Ratio of EtOH	Ratio of water	Batch Yield (%)	Space Time to Yield (kg m^{-3} day^{-1})
CAU-10H	1[a]	1	0	3	180	93	140
Al-75IPA-25PYDC	1[b]	0.75	0.25	0	300	85	65.7
Al-50IPA-50PYDC	1[b]	0.5	0.5	0	300	83	64.1
Al-25IPA-75PYDC	1[b]	0.25	0.75	0	300	83	64.1
CAU-10pydc	1[b]	0	1	0	300	93	72.0

[a] The Al source was $Al_2(SO_4)_3·18H_2O$ and $NaAlO_2$

[b] The Al source was $AlCl_3·6H_2O$

Table S2. Structure formula of the Al-xIPA-(100-x)PYDC samples calculated and compared by elemental analysis (EA), inductively coupled plasma-atomic emission spectroscopy (ICP-AES) and ^{1}H NMR experimental data.

**Compound**	**Alumium wt%**	**Carbon wt%**	**Hydrogen wt%**	**Nitrogen wt%**	**Formula by EA and ICP**	**Ratio of linkers IPA/PDC by ^{1}H NMR**
**Al-25IPA-75PYDC**	12.1	40.2	2.4	4.5	$Al(OH)IPA_{0.3}PDC_{0.7}\cdot H_2O_{0.18}$	0.27/0.73
**Al-50IPA-50PDC (KMF-2)**	12.0	41.6	2.3	2.9	$Al(OH)IPA_{0.54}PDC_{0.46}\cdot H_2O_{0.30}$	0.53/0.47
**Al-75IPA-25PDC**	12.3	43.4	2.7	1.4	$Al(OH)IPA_{0.78}PDC_{0.22}\cdot H_2O_{0.26}$	0.78/0.22

Table S3. Textural features of KMF-2 (pristine and after hydrothermal and chemical stability test) and other synthesized compounds calculated from N_2 physisorption measurements at −195.8 °C.

**Sample**	S_{BET} (m^2 g^{-1})[c]	S_{micro} (m^2 g^{-1})[d]	$V_{p, total}$ (cm^3 g^{-1})	$V_{p, micro}$ (cm^3 g^{-1})[d]
CAU-10pydc	1030	998	0.420	0.391
Al-25IPA-75PYDC	889	820	0.361	0.291
Al-50IPA-50PYDC (KMF-2)	819	788	0.334	0.287
Al-75IPA-25PDC	698	591	0.331	0.208
CAU-10-H	670	631	0.260	0.233
KMF-2 after hydrothermal test [a]	819	751	0.307	0.275
KMF-2 (pH=1) [b]	815	714	0.327	0.260
KMF-2 (pH=2) [b]	826	734	0.320	0.266
KMF-2 (pH=10) [b]	812	760	0.280	0.279
KMF-2 (pH=11) [b]	823	743	0.313	0.273
KMF-2 (pH=12) [b]	808	697	0.342	0.251

[a]The data was collected after boiling water treatment for 24 h.

[b]The data was collected after immersed in various acidic and basic aqueous solutions (pH 1–12).

[c]Specific surface area (S_{BET}) was calculated using the Brunauer–Emmett–Teller (BET) equation.

[d]The micropore surface area (S_{micro}) and volume (V_{micro}) was calculated using the *t*-plot method.

Table S4. Crystallographic parameters for KMF-2 hydrated obtained from the Rietveld refinement.

	**KMF-2 hydrated**	**KMF-2 dehydrated**
Refined unit cell composition	C_{120} Al_{16} N_8 $O_{118.276}$	C_{120} Al_{16} $N_{22.403}$ O_{80}
Symmetry	Tetragonal	Tetragonal
Space group	*I -4 2 d*	$I\ 4_1\ 2\ 2$
a (Å)	21.527(4)	21.527(5)
c (Å)	10.507(2)	10.368(2)
Cell volume (Å^3)	4869.2(18)	4805(2)
λ (Å)	1.54187	1.54187
Number of points	5250	5250
Number of parameters	25	36
Number of restraints	0	0
Number of constraints	1	0
ls_shift/su_max	0.0623	0.0628
diff_density_max	0.39	0.21
diff_density_min	-0.83	-0.39
R_p(%)	6.51	8.83
R_{wp}(%)	8.76	11.29
R_F(%)	8.11	6.09
GOF	0.79	1.03

Table S5. Experimental data on Rietveld refinement of KMF-2.

Atom	Type	x	y	z	Uiso(Å^2)	Multiplicity	Occupancy
Al1	Al	0.178(3)	0.25	0.125	0.017(10)	8	1
O1	O	0.237(4)	0.763(4)	0	0.003(18)	8	1
Al2	Al	0.813(3)	0.25	0.125	0.017(10)	8	1
O2	O	0.244(4)	0.244(4)	0	0.003(18)	8	1
C1	C	0.6789(11)	0.5469(19)	0.7872(19)	0.108(15)	16	1
C2	C	0.6985(9)	0.5531(17)	0.669(2)	0.108(15)	16	1
C3	C	0.7092(6)	0.5007(18)	0.6051(15)	0.108(15)	16	1
C4	C	0.6962(9)	0.4450(17)	0.662(2)	0.108(15)	16	1
C5	C	0.6719(11)	0.4477(19)	0.786(2)	0.108(15)	16	1
C6	C	0.6639(11)	0.498(2)	0.8431(16)	0.108(15)	16	0.5
N6'	N	0.6639(11)	0.498(2)	0.8431(16)	0.108(15)	16	0.5
C7	C	0.7052(15)	0.3921(17)	0.602(3)	0.108(15)	16	1
O3	O	0.7377(17)	0.388(2)	0.496(3)	0.108(15)	16	1
C8	C	0.7096(15)	0.6098(17)	0.617(3)	0.108(15)	16	1
O4	O	0.7557(18)	0.623(2)	0.542(4)	0.108(15)	16	1
O5	O	0.665(2)	0.6583(18)	0.617(4)	0.108(15)	16	1
O6	O	0.684(2)	0.3350(17)	0.653(4)	0.108(15)	16	1
				**Guest species**			
N1	N	0.611(6)	0.511(13)	1.008(15)	0.09(7)	16	0.4721
N2	N	0.568(7)	0.454(7)	1.218(16)	0.09(7)	16	0.4281

Table S6: DFT-electronic energy, cell parameters and textural properties associated with the 4 distinct DFT-optimized KMF-2 configurations represented in **Figure S16**.

	**KMF-2_1**	**KMF-2_2**	**KMF-2_3**	**KMF-2_4**
ΔE_{cell}(kJ/mol)	0	7.16883	0.739451	7.84295
a (Å)	21.638	21.643	21.645	21.641
b (Å)	21.624	21.642	21.643	21.644
c (Å)	10.340	10.261	10.262	10.273
$\alpha = \beta = \gamma$ (^{o})	90	90	90	90
Cell volume (Å^3)	4838.09	4806.23	4807.36	4811.85
Density (g/cm^3)	1.14553	1.15313	1.15285	1.15178
Pore volume (cm^3/g)	0.455	0.447	0.449	0.450
Pore limiting Diameter (Å)	6.15	6.10	6.10	6.10
N2-accessible Surface area (m^2/g)	830	800	800	815

Table S7. Temperature at the highest desorption rate (T_d) under various heating rates (β) and activation energy (E_d) of CAu-10H, KMF-2, and CAU-10pydc.

**Material**	**T_d (ºC) under various β (ºC min^{-1})**					**E_d (kJ mol^{-1})**
	4	6	8	10	12	
CAU-10H	75.5	79.3	82.6	85.8	88.3	83
KMF-2	81.0	84.9	88.1	90.9	93.7	88
CAU-10pydc	89.9	93.5	96.9	99.7	102.7	91

Table S8. Water sorption properties and energy storage capacities of KMF-2, the platform MOFs and benchmark water adsorbents.

Material	Crystal density (g/cm^3)	Working capacity[a]		Specific energy capacity[b]		Energy storage capacity[c]	
		($g{\cdot}g^{-1}$)	($mL{\cdot}mL^{-1}$)	($Wh{\cdot}kg^{-1}$)	($kWh{\cdot}m^{-3}$)	($Wh{\cdot}kg^{-1}$)	($kWh{\cdot}m^{-3}$)
KMF-2	1.15	0.30[d]	0.34[d]	205.9 (214.7)	236.7 (246.9)	286	329
CAU-10pydc	1.18[d]	0.12[d]	0.14[d]	86.4 (93.7)[d]	102.2 (110.8)[d]	130[d]	154[d]
CAU-10H	1.15[d]	0.26[d]	0.30[d]	185.3 (189)[d]	213 (217.4)[d]	238[d]	274[d]
MIP-200	1.16[d]	0.14[d]	0.16[d]	88.4 (150.2)[d]	102.5 (174.2)[d]	189[d]	220[d]
Co-CUK-1	1.46[d]	0.24[d]	0.35[d]	173.6 (180.7)[d]	253.4 (263.8)[d]	207[d]	302[d]
MOF-303	1.012[d]	0.254[d]	0.257[d]	178.3 (188.0)[d]	180.4 (190.2)[d]	310[d]	258[d]
MIL-160	1.068[d]	0.11[d]	0.12[d]	67.7 (74.7)[d]	72.3 (79.8)[d]	108[d]	115[d]
KMF-1	1.080[d]	0.33	0.36	243.7 (246.3)	263.2 (266)	323[d]	348[d]
CAU-23	1.07[d]	0.016[d]	0.017[d]	8.23 (136.6)[d]	8.82 (146.2)[d]	168[d]	180[d]
SAPO-34	1.43[d]	0.10[d]	0.14[d]	115 (120)[d]	165 (172)[d]	115[d]	165[d]

[a] Working capacity deduced from one refrigeration cycle at T_{ev} = 5 °C, T_{ads} = 30 °C, T_{con} = 30 °C, and T_{des} = 70 °C;

[b] Heat transferred from the evaporator in one refrigeration cycle at T_{ev} = 5 °C, T_{ads} = 30 °C, T_{con} = 30 °C, and T_{des} = 70 °C (T_{ev} = 10 °C for values in parentheses);

[c] Energy storage capacity per unit weight or volume of adsorbent at T_{ev} = 10 °C, T_{ads} = 30 °C, T_{con} = 30 °C, and T_{des} = 70 °C;

[d] Values of crystal density, working capacity, heat from evaporator, and heat storage capacity for MIP-200,[12] Co-CUK-1,[13] CAU-10-H,[14] MOF-303,[15] SAPO-34,[7] MIL-160,[16] CAU-23[17] and KMF-1[18] were calculated by characteristic curves and data taken from references.

Table S9. The prices of typical starting material for synthesis of MOF water adsorbents.

Chemicals	CAS No.	Price ($/g)	Supplier
$AlCl_3 \cdot 6H_2O$	7784-13-6	76.1 $ / 500 g	Sigma-Aldrich co. ltd
$Al_2(SO_4)_3 \cdot 18H_2O$	7784-31-8	736 $ / 2,500 g	Sigma-Aldrich co., ltd
$Al(OH)(OAc)_2$	142-03-0	213 $ / 500 g	Sigma-Aldrich co., ltd
$ZrCl_4$	10026-11-6	243 $ / 500 g	Sigma-Aldrich co., ltd
$CoCl_2 \cdot 6H_2O$	7791-13-1	255 $ / 500 g	Sigma-Aldrich co., ltd
3,5-Pyridinedicarboxylic acid	499-81-0	49 $ / 25 g	Tokyo Chemical Industry co., ltd
Isophthalic acid	121-91-5	25 $ / 500 g	Tokyo Chemical Industry co., ltd
2,5-Furandicarboxylic acid	3238-40-2	179 $ / 25 g	Tokyo Chemical Industry co., ltd
1H-2,5-Pyrroledicarboxylic acid	937-27-9	580 $ / 1 g	CHEMSOON co. ltd.
3,5-Pyrazoledicarboxylic acid	303180-11-2	213 $ / 25 g	Tokyo Chemical Industry co., ltd
3,3′,5,5′-Tetracarboxydiphenylmethane	10397-52-1	2125 $ / 5 g	CHEMSOON co. ltd.
2,4-Pyridinedicarboxylic acid	499-80-9	195 $ / 25 g	Tokyo Chemical Industry co., ltd
2,5-Thiophenedicarboxylic acid	4282-31-9	251 $/25 g	Sigma-Aldrich co., ltd

Table S10. Material cost for producing MOF water adsorbents.

MOF adsorbent	Metal precursor	Organic linker	Reaction yield (%)	Material cost[a] ($/g)	References
KMF-2	$AlCl_3 \cdot 6H_2O$	Isophthalic acid<br>3,5-Pyridinedicarboxylic acid<br>(50:50)	83	1.18	**This work**
CAU-10pydc	$AlCl_3 \cdot 6H_2O$	3,5-Pyridinedicarboxylic acid	93	1.87	[19]
CAU-10H	$Al_2(SO_4)_3 \cdot 18H_2O$<br>$NaAlO_2$	Isophthalic acid	93	0.43	[14]
MIL-160	$Al(OH)(OAc)_2$	2,5-Furandicarboxylic acid	93	6.44	[16]
KMF-1	$Al_2(SO_4)_3 \cdot 18H_2O$	1H-2,5-Pyrroledicarboxylic acid	93	972.68	[18]
MOF-303	$AlCl_3 \cdot 6H_2O$	3,5-Pyrazoledicarboxylic acid	23	28.23	[15]
MIP-200	$ZrCl_4$	3,3′,5,5′-Tetracarboxydiphenylmethane	96	172	[12]
Co-CUK-1	$CoCl_2 \cdot 6H_2O$	2,4-Pyridinedicarboxylic acid	67	10.54	[13]
CAU-23	$AlCl_3 \cdot 6H_2O$[b]	2,5-Thiophenedicarboxylic acid	84	9.81	[17]

[a] Material cost was calculated by using the required amounts of metal precursor and ligand precursor for obtaining 1g of adsorbent excluding not only the prices related to starting materials such as solvent, modulator, and additives but also the operation and utility cost for hydrothermal reaction, filtrate, and drying etc.

[b] The actual metal precursor is solution of $AlCl_3$. For calculation, the cheapest solid salt ($AlCl_3 \cdot 6H_2O$) is selected.

References

[1] J. P. Perdew, K. Burke, M. Ernzerhof, *Phys. Rev. Lett.* **1996,** *77* (18), 3865.

[2] S. Goedecker, M. Teter, J. Hutter, *Physical Review B* **1996,** *54* (3), 1703.

[3] J. VandeVondele, J. Hutter, *J. Chem. Phys.* **2007,** *127* (11), 114105.

[4] S. Grimme, J. Antony, S. Ehrlich, H. Krieg, *J. Chem. Phys.* **2010,** *132* (15), 154104.

[5] T. A. Manz, D. S. Sholl, *J. Chem. Theory Comput.* **2010,** *6* (8), 2455.

[6] A. K. Rappé, C. J. Casewit, K. Colwell, W. A. Goddard III, W. M. Skiff, *J. Am. Chem. Soc.* **1992,** *114* (25), 10024.

[7] M. F. de Lange, K. J. Verouden, T. J. Vlugt, J. Gascon, F. Kapteijn, *Chem. Rev.* **2015,** *115* (22), 12205.

[8] J. L. Abascal, C. Vega, *J. Chem. Phys.* **2005,** *123* (23), 234505.

[9] T. J. Bandosz, J. Jagiełło, J. A. Schwarz, *J. Chem. Eng. Data* **1996,** *41* (4), 880.

[10] H. E. Kissinger, Anal. Chem. 1957, 29 (11), 1702.

[11] X. Liu, X. Wang, F. Kapteijn, *Chem. Rev.* **2020,** *120* (16), 8303.

[12] S. Wang, J. S. Lee, M. Wahiduzzaman, J. Park, M. Muschi, C. Martineau-Corcos, A. Tissot, K. H. Cho, J. Marrot, W. Shepard, G. Maurin, J.-S. Chang, C. Serre, *Nat. Energy* **2018,** *3* (11), 985.

[13] J. S. Lee, J. W. Yoon, P. G. Mileo, K. H. Cho, J. Park, K. Kim, H. Kim, M. F. de Lange, F. Kapteijn, G. Maurin, *ACS Appl. Mater. Interfaces* **2019,** *11* (29), 25778.

[14] D. Lenzen, P. Bendix, H. Reinsch, D. Fröhlich, H. Kummer, M. Möllers, P. P. Hügenell, R. Gläser, S. Henninger, N. Stock, *Adv. Mater.* **2018,** *30* (6), 1705869.

[15] N. Hanikel, M. S. Prévot, F. Fathieh, E. A. Kapustin, H. Lyu, H. Wang, N. J. Diercks, T. G. Glover, O. M. Yaghi, *ACS Cent. Sci.* **2019,** *5* (10), 1699.

[16] A. Cadiau, J. S. Lee, D. Damasceno Borges, P. Fabry, T. Devic, M. T. Wharmby, C. Martineau, D. Foucher, F. Taulelle, C. H. Jun, *Adv. Mater.* **2015,** *27* (32), 4775.

[17] D. Lenzen, J. Zhao, S. J. Ernst, M. Wahiduzzaman, A. Ken Inge, D. Frohlich, H. Xu, H. J. Bart, C. Janiak, S. Henninger, G. Maurin, X. Zou, N. Stock, *Nat. Commun.* **2019,** *10* (1), 3025.

[18] K. H. Cho, D. D. Borges, U. Lee, J. S. Lee, J. W. Yoon, S. J. Cho, J. Park, W. Lombardo, D. Moon, A. Sapienza, *Nat. Commun.* **2020,** *11* (1), 1.

[19] K. H. Cho, D. D. Borges, J. S. Lee, J. Park, S. J. Cho, D. Jo, U. H. Lee, G. Maurin, J.-S. Chang, *ACS Sustainable Chem. Eng.* **2022,** *10* (21), 7010.
